# Genotypic similarities among the parthenogenetic *Darevskia* rock lizards with different hybrid origins

**DOI:** 10.1186/s12862-020-01690-9

**Published:** 2020-09-16

**Authors:** David Tarkhnishvili, Alexey Yanchukov, Mehmet Kürşat Şahin, Mariam Gabelaia, Marine Murtskhvaladze, Kamil Candan, Eduard Galoyan, Marine Arakelyan, Giorgi Iankoshvili, Yusuf Kumlutaş, Çetin Ilgaz, Ferhat Matur, Faruk Çolak, Meriç Erdolu, Sofiko Kurdadze, Natia Barateli, Cort L. Anderson

**Affiliations:** 1 Institute of Ecology, Ilia State University, Tbilisi, Georgia; 2 Zonguldak Bülent Ecevit University, Zonguldak, Turkey; 3 Faculty of Science, Department of Biology, Hacettepe University, Ankara, Turkey; 4 Faculty of Science, Department of Biology, Dokuz Eylül University, İzmir, Turkey; 5 Moscow State University, Moscow, Russia; 6 Yerevan State University, Yerevan, Armenia; 7 Middle East Technical University, Faculty of Science, Department of Biology, Ankara, Turkey

**Keywords:** Darevskia, Parthenogenesis, Microsatellites, Mitochondrial DNA, Backcrosses, Allele conversion, Caucasian rock lizards

## Abstract

**Background:**

The majority of parthenogenetic vertebrates derive from hybridization between sexually reproducing species, but the exact number of hybridization events ancestral to currently extant clonal lineages is difficult to determine. Usually, we do not know whether the parental species are able to contribute their genes to the parthenogenetic vertebrate lineages after the initial hybridization. In this paper, we address the hypothesis, whether some genotypes of seven phenotypically distinct parthenogenetic rock lizards (genus *Darevskia*) could have resulted from back-crosses of parthenogens with their presumed parental species. We also tried to identify, as precise as possible, the ancestral populations of all seven parthenogens.

**Results:**

We analysed partial mtDNA sequences and microsatellite genotypes of all seven parthenogens and their presumed ansectral species, sampled across the entire geographic range of parthenogenesis in this group. Our results confirm the previous designation of the parental species, but further specify the maternal populations that are likely ancestral to different parthenogenetic lineages. Contrary to the expectation of independent hybrid origins of the unisexual taxa, we found that genotypes at multiple loci were shared frequently between different parthenogenetic species. The highest proportions of shared genotypes were detected between (i) *D. sapphirina* and *D. bendimahiensis* and (ii) *D. dahli* and *D. armeniaca*, and less often between other parthenogens. In case (ii), genotypes at the remaining loci were notably distinct.

**Conclusions:**

We suggest that both observations (i-ii) can be explained by two parthenogenetic forms tracing their origin to a single initial hybridization event. In case (ii), however, occasional gene exchange between the unisexual and the parental bisexual species could have taken place *after* the onset of parthenogenetic reproduction. Indeed, backcrossed polyploid hybrids are relatively frequent in *Darevskia*, although no direct evidence of recent gene flow has been previously documented. Our results further suggest that parthenogens are losing heterozygosity as a result of allelic conversion, hence their fitness is expected to decline over time as genetic diversity declines. Backcrosses with the parental species could be a rescue mechanism which might prevent this decline, and therefore increase the persistance of unisexual forms.

## Background

Parthenogenesis has both advantages and shortcomings compared to sexual reproduction [1–10]. One significant advantage of parthenogenesis relative to sexual reproduction is the absence of males, which allows the allocation of more resources into the production of offspring [5, 6]. On the other hand, the lack of genetic recombination leads to an accumulation of deleterious mutations in the genome of parthenogens, whereas in sexual breeders, recombination and natural selection can eliminate recessive deleterious mutations [8, 11, 12]. All this, coupled with the lack of novel recombinant genotypes, dramatically reduces the ability of the parthenogens to adapt to changes in the environment [5, 7, 10]. Occasional true sex, even if it occurs only once in many thousands of generations, solves these problems; therefore, it is an effective reproduction strategy [8]. The fact that parthenogenesis with occasional true sex is found throughout the tree of life, being pervasive in plants, prokaryotes, protists, insects, and multiple other phyla [1, 13–18], supports the advantage of this form of reproduction.

Unlike plants and invertebrates, parthenogenesis in vertebrates is rare, and parthenogenetic vertebrates are usually unable to enrich their genomes by occasional sex. There are only a few exceptions: 1) A gynogenetic Amazon molly fish (*Poecilia formosa*) exhibits recombination, which has occurred after the initial hybridization [19, 20]. 2) The North American salamanders, *Ambystoma*, are also gynogenetic and can incorporate genetic elements received horizontally from closely related sexual species [21]. Both systems appear to be among the oldest vertebrate parthenogenetic lineages, with an estimated age of ~ 5 Mye [20, 22].

In all known cases of unisexual species of reptiles, parthenogenesis appears to be obligate [23], and no gene exchange with sexually reproducing species has been documented (although hypothesized [24]). Parthenogenetic forms are most common among the squamates (19 genera [25]). For all but one of these genera, parthenogenesis results from interspecific hybridization [25, 26]. Consequently, such unisexual species initially possess highly heterozygous genotypes, and “freezing” this high initial diversity enables remarkable evolutionary persistence of many parthenogenetic vertebrates [23, 27, 28]. In some groups, hybrid parthenogens are diverse and occupy large geographic areas: the North American whiptail lizards (*Aspidoscelis*) and Caucasian Rock Lizards (*Darevskia*) have produced multiple unisexual lineages in parallel [29–37].

**Fig. 1:**
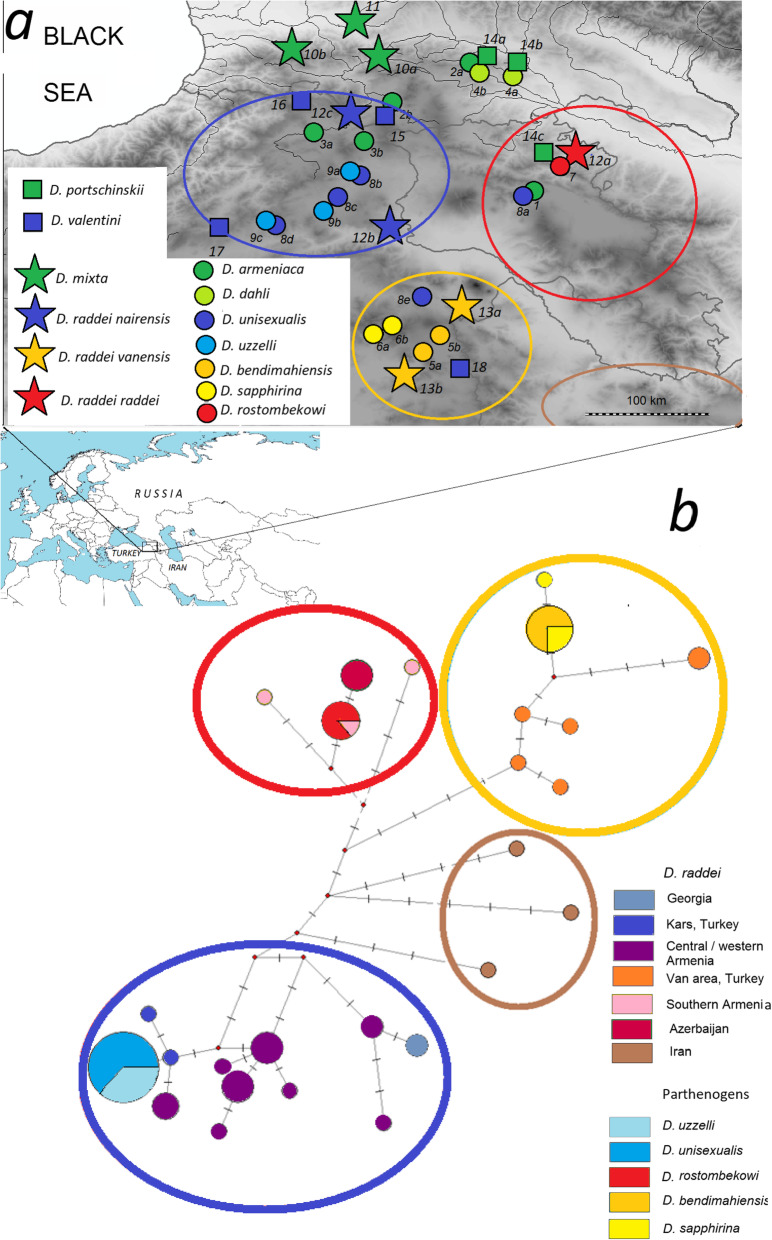
Map of sampling locations (**a**) and Median Joining network linking *D. raddei* and its daughter parthenogenetic forms (**b**). Location numbers (same as in Table 3) are shown on the map. Parental species in boldface. *D. armeniaca*: 1 - Hrazdan, Armenia; 2a - Didgori, Georgia; 2b - Khospio, Georgia; 3a - Ardahan, Turkey; 3b - Çıldır, Turkey; *D. dahli*: 4a – Kojori, Georgia; 4b – Didgori, Georgia; *D. bendimahiensis*: 5a - Muradiye, Turkey; 5b - Çaldıran, Turkey; *D. sapphirina*: 6a - Patnos, Turkey; 6b - Pınarlı, Turkey; *D. rostombekowi*: 7 - Dilijan, Armenia; *D. unisexualis*: 8a - Hrazdan, Armenia; 8b - Hanak, Turkey; 8c- Horasan, Turkey; 8d – River Ağrı, Turkey; *D. uzzelli*: 9a – Kars, Turkey; 9b - Sarıkamış, Turkey; 9c – Horasan, Turkey; *D. mixta*: 10a – Akhaldaba, Georgia; 10b – Abastumani, Georgia; 11 – Ambrolauri, Georgia; *D. raddei raddei*: 12а - Sotk Village, Armenia; *D. raddei nairensis*: 12b - Digor, Turkey; 12c - Vardzia, Georgia; *D. raddei vanensis*: 13a - Doğubeyazıt, Turkey; 13b - Muradiye, Turkey. *D. portschinskii*: 14a - River Khrami, Georgia; 14b - Kojori, Georgia; 15 - Mets Sepasar, Armenia; *D. valentini*: 15 – Akhalkalaki, Georgia; 16 - Ardahan, Turkey; 17 - Erzurum, Turkey; 18 - Çaldıran, Turkey. Circles of the same color are delimiting sampling areas in (**a**) and related haplogroups (**b**). Brown circle delimits haplogroups of *D. raddei* from Iran downloaded from GenBank, unrelated to any known parthenogenetic form, and hence not included in further analyses. The Caucasus Map was prepared in software QGIS (http://qgis.osgeo.org). Eurasia’s map at the bottom of the figure is modified from World Map Blank.svg (Public domain)

Caucasian rock lizards are the first group of vertebrates where parthenogenesis was discovered. Lantz & Cyren [38] were among the first who noted the absence of males in some populations of rock lizard *Lacerta saxicola armeniaca* (later designated *Darevskia armeniaca*), from Armenia. Darevsky [39] documented locations where only females were present and suggested they were obligate parthenogens. In his 1967 treatise, he described four parthenogenetic forms (subsequently given species status) from Armenia and Georgia [40]: *D. armeniaca, D. dahli, D. unisexualis,* and *D. rostombekowi*. Later, another species from Turkey was discovered, *D. uzzelli* [41]. Finally, two new parthenogenetic forms, *D. sapphirina* and *D. bendimahiensis* were described from the vicinity of Lake Van in Turkey [42]. All these parthenogens live in the area between the Lesser Caucasus Mountains and Lake Van (Fig. 1).

All parthenogenetic *Darevskia* descend from hybridization between bisexual representatives of two clades within the genus: the maternal ancestry comes exclusively from the *caucasica* clade (*D. mixta, D. raddei*) and the paternal ancestors from the *rudis* clade (*D. valentini, D. portschinskii*). These two clades separated between ten [35] and 25 million years ago [43, 44]. *D. armeniaca* and *D. dahli* share mitochondrial DNA descended from the Western Caucasian *D. mixta*. Five other unisexuals: *D. unisexualis, D. rostombekowi, D. uzzelli, D. bendimahiensis,* and *D. sapphirina* descended matrilineally from at least two distinct populations of *D. raddei,* currently found south of the Lesser Caucasus Mountains [36, 45]. *D. mixta* and *D. raddei*, although they belong to the same clade within *Darevskia*, are genetically distinct, and they are not even sister species; hence the parthenogens matrilineally associated with *D. raddei* and *D. mixta* are also distinctive, and their mitochondrial haplogroups are reciprocally monophyletic [45].

The details of patrilineal ancestry are less clear. The bisexual species *D. portschinskii* shares the highest proportion of allozyme alleles with *D. dahli* and *D. rostombekowi*, and is partially sympatric with both of them in the eastern Lesser Caucasus. *D. valentini* from the higher altitudes south and west of the Lesser Caucasus has the highest number of allozyme alleles shared with five other parthenogens [45]. The use of small sample sizes genotyped for allozyme markers with low allelic diversity was a significant limitation in the latter study, and did not allow detailed resolution of a reticulate speciation scheme (Fig. 1 in [45]). Disentangling the paternal ancestral contribution is further complicated because, unlike *D. mixta* and *D. raddei* on the maternal side, *D. portschinkii* and *D. valentini* exchange genes via introgression between themselves and with other species within the *rudis* clade [46].

For decades, the number of hybridization events that led to a series of parthenogenic forms in *Darevskia* has been subject to debate. Parker et al. [47] did not exclude the occasional presence of more than one clonal hybrid lineage within the same taxon. Later publications also indirectly suggest polyclonality: Fu et al. [48] discovered allozyme variation within *D. unisexualis* (three genotypes), *D. uzzelli,* and *D. bendimahiensis* (two genotypes each). Polymorphisms were also recorded for *D. armeniaca* [36, 49, 50], *D. dahli* [51, 52], *D. unisexualis* [37, 48], *D. rostombekowi* [53], *D. uzzelli* and *D. bendimahiensis* [48]. Vergun et al. [54], Ryskov et al. [53], and Girnyk et al. [55] examined the nucleotide sequence variation in amplicons containing the microsatellite repeats, in *D. dahli*, *D. rostombekowi*, and *D. armeniaca*. The variation in the repeat lengths at individual loci was attributed to mutations within the clonal parthenogenetic lineages, while the single-nucleotide variants in the regions flanking the repeats were assumed to be due to independent hybridization events. However, no formal model-based criteria were applied to distinguish between multiple hybridizations vs mutation accumulation scenarios, which cannot be excluded a *priori* [54, 56, 57].

Although the possibility of ongoing gene flow from a parental species into the parthenogenetic forms of reptiles is usually not considered, it can not be excluded completely [24]. Darevsky and Kulikova [58] mentioned the presence of hybrids between parthenogenetic *D. armeniaca* and sexually reproducing *D. valentini*; all of them were triploid, and females were sterile [58–60], although later some triploids with fully developed gonads were found [61–63]. Freitas et al. [37] genotyped multiple triploid individuals in sympatric populations of *D. valentini, D. armeniaca,* and *D. unisexualis*, but ruled out gene introgression from the presumed paternal species into parthenogenetic lineages. Triploid individuals were also found where parthenogenetic *D. dahli* and *D. armeniaca* are sympatric with *D. raddei* in Armenia [60, 64, 65].

Tarkhnishvili et al. [24] studied multiple genotypes at five microsatellite loci in *D. armeniaca, D. dahli*, their maternal ancestor *D. mixta*, and anticipated paternal ancestors *D. portschinskii* and *D. valentini*. The majority of individuals of both parthenogens had coincident genotypes at two loci and different most-frequent genotypes at the three other loci. The authors concluded that the most plausible explanation of this pattern is rare backcrossing that leads to the integration of parts of a different paternal genome into the genome of existing parthenogenetic form; hence, *D. armeniaca* is a result of backcross between parthenogenetic *D. dahli* with *D. valentini*, and not a hybrid between *D. valentini* and *D. mixta*.

In summary, unexpectedly high levels of genetic variation, and apparent post-hybridization reorganization and redistribution of alleles in *Darevskia* contradict the standard theory, which predicts relative genomic stasis. Coinciding microsatellite genotypes in *D. dahli* and *D. armeniaca* and high genetic diversity within these and other unisexual forms raise questions on the possible role of interbreeding between parthenogenetic and bisexual lizards in the diversification of parthenogenetic *Darevskia* (and, perhaps, other groups of parthenogenetic lizards).

In the present study, we aimed to clarify some important questions about the origin and population genetic structure of parthenogenetic *Darevskia*. We analyze the microsatellite genotypes and mitochondrial haplotypes in all seven parthenogenetic species of rock lizards from Turkey, Georgia, and Armenia, as well as in their presumed parental populations. We attempt to identify, as precisely as possible, maternal and paternal source populations of each parthenogen. While several studies with similar goals were conducted earlier [36, 45], including a recent one [37], none of them included all parthenogenetic *Darevskia* species*¸* nor did they sample the ranges of the ancestral species on the scale presented here. Importantly, we examined the evidence for (i) a single initial hybridization event in the origin of each parthenogenetic *Darevskia*, a hypothesis shared by most authors [35, 37, 45] and (ii) an alternative hypothesis, that posits new parthenogenetic forms may have resulted from the backcross of a hybrid parthenogen with a paternal bisexual species [24]. Our results suggest that the origin and evolution of the parthenogenetic forms of *Darevskia* are far more complicated than considered earlier. The genetic similarities between some parthenogenetic lineages can hardly be explained solely by coincidence. Simultaneously, we exclude the multiclonal origin of any of the described parthenogenetic species of *Darevskia*.

## Results

### Matrilineal ancestry of the parthenogens

**Fig. 2:**
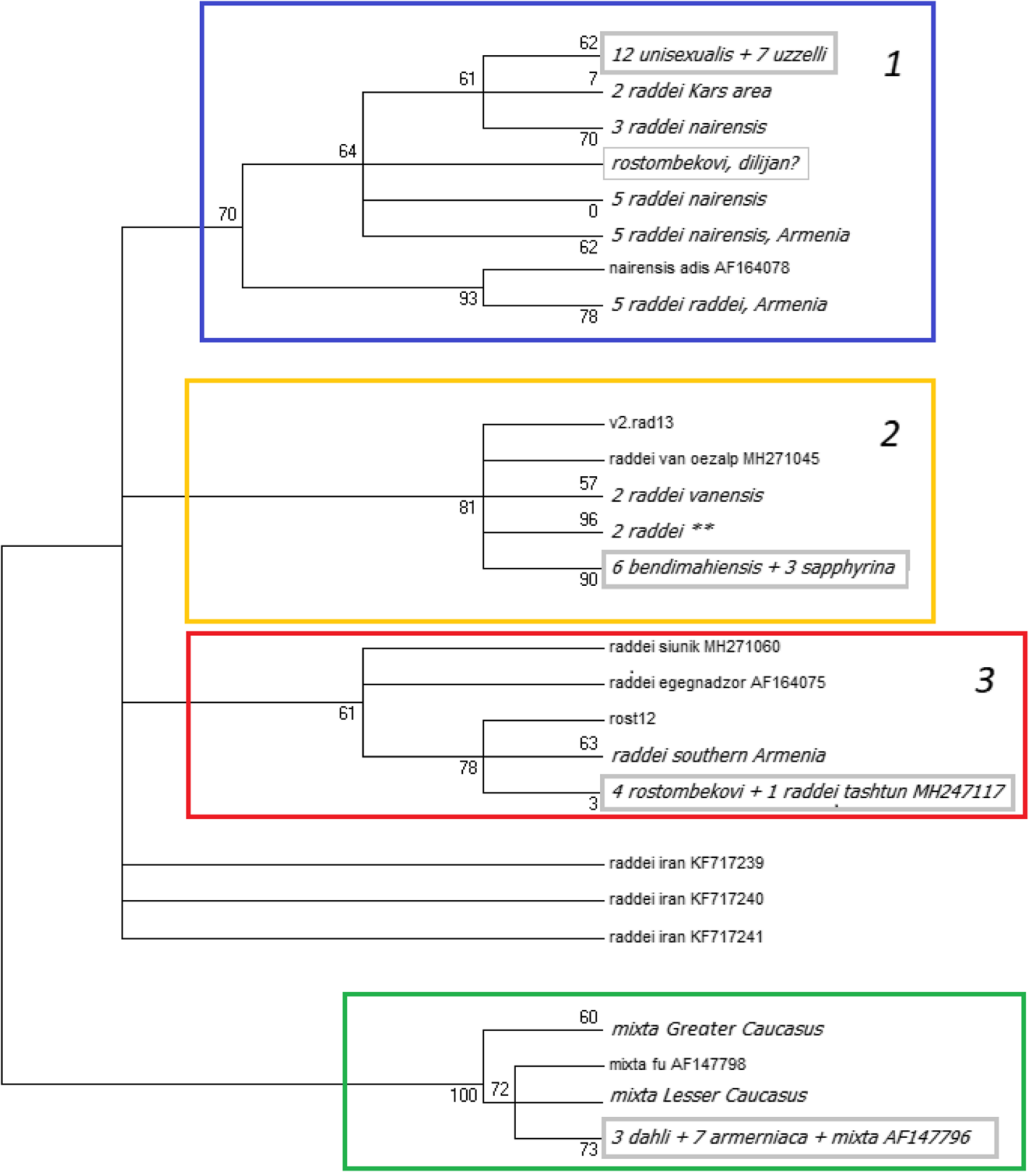
Maximum Likelihood 50% consensus rule tree of haplotypes associated with different populations *D. raddei* and five its parthenogenetic daughter species, based on the HKY + G model. The 683 bp fragment of the mitochondrial *Cytochrome b* gene is used. The tree with the highest log likelihood (− 799.7117) is shown. The tree is based on 42 novel sequences and 62 sequences downloaded from GenBank. Bootstrap values exceeding 50% are shown on the nodes and branches. Individual clades are shown in color boxes. Clade 1 (blue box) *- D*. *r. nairensis* and *D. r. raddei* from the northernmost part of the species range and their daughter parthenogenetic species; clade 2 (yellow box) – *D. r. vanensis* and its daughter parthenogenetic species. Clade 3 (red box) – *D. r. raddei* from southern Armenia and Azerbaijan and their daughter parthenogenetic species; *D. mixta* and its daughter parthenogens (green box) are used as an outgroup for *D. raddei* and its daughter parthenogens. For the relative position of *D. raddei* and *D. mixta* in the phylogenetic tree of *Darevskia,* see [45, 66]

The best-fit substitution model (that with the lowest BIC score) for all samples of the parthenogens and their presumed ancestral species was HKY + G, assuming variable base frequencies, stable transition rates, and stable transversion rates. The Maximum Likelihood tree (Fig. 2) based on the analysis of 683 bp of mitochondrial cytochrome b gene, both of the novel samples and those downloaded from GenBank showed the presence of three well-supported clades within *D. raddei* and its daughter parthenogens: 1) *D. raddei* from southern Georgia and the Kars area in Turkey (nominal subspecies *D. r. nairensis* and *D. r. raddei*), which also includes all individuals of *D. unisexualis* and *D. uzzelli*. One sequence from GenBank marked as “*D. rostombekovi*” (GenBank #MH247113) was also clustered with this clade. However, this might be a result of an error in species identification, since all other *D. rostombekovi* belong to a different clade. This sequence was excluded from the rest of the analyses.) 2) a clade including *D. raddei vanensis, D. bendimahiensis,* and *D. sapphirina;* and 3) a clade including individuals from southern Armenia and Azerbaijan, which also includes *D. rostombekowi* (colored boxes in Fig. 2).

The published sequences of *D. raddei* from Iran belonged to three additional lineages in a polytomy. The individuals of *D. mixta* were from both the Greater and the Lesser Caucasus lineages [67] and all *D. armeniaca* and *D. dahli* are clustered with the Lesser Caucasus clade.

The median-joining network of all individuals (Fig. 1b) revealed 24 haplotypes of *D. raddei,* seven of *D. mixta,* and seven of their daughter species. Six for those descending from the maternal lineages of *D. raddei* and one of *D. mixta* (a previous study that analyzed shorter sequences of many more *D. dahli* and *D. armeniaca* revealed the presence of multiple haplotypes of these species, although the majority were identical to that described in [24]). There is little variation among individuals of the same parthenogenetic form. Simultaneously, (1) most of the individuals of *D. armeniaca* and *D. dahli* share the same haplotype, which is also present in a single individual of *D. mixta* from Borjomi Gorge (see also [24]; (2) all individuals of *D. uzzelli* and *D. unisexualis* share the same haplotype, which is close to the haplotypes of *D. raddei* from Digor near Kars, Turkey (subspecies *D. r. nairensis*); (3) all *D. rostombekowi* share the same haplotype, which is also recorded in *D. raddei* from Tashtun in Southern Armenia (subspecies *D. r. raddei*); (4) all *D. bendimahiensis* samples, and all but one *D. sapphirina* share a common haplotype closest to the haplotype of *D. raddei* from Doğubeyazıt and Özalp in the Lake Van area, Turkey (subspecies *D. r. vanensis*, Fig. 1b). In summary, parthenogens that matrilineally descend from *D. raddei* stem from at least three geographically distinct lineages of this species.

### Genetic diversity of parthenogens and their putative ancestors

**Fig. 3:**
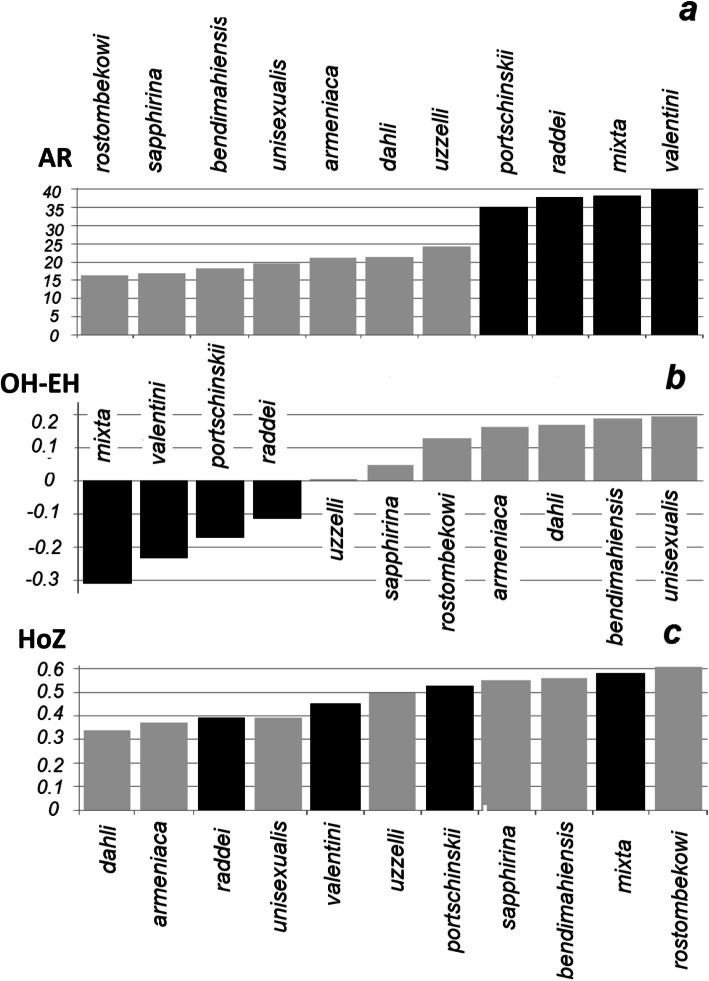
**a** Allelic richness (the value of the index of Petit et al. [67]) (AR); **b** the difference between the observed and expected heterozygosity (OH – EH); **c** and the overall proportion of homozygous alleles (HoZ). Parthenogenetic taxa (grey) and their presumed ancestral species (black)

The entire data set of microsatellite genotypes is presented in Table S1. As expected, genetic diversity of all parthenogenetic forms is lower than in their putative paternal and maternal species; the average allelic richness [68] in the parthenogens is 1.9 times lower than in their presumed ancestors (Fig. 3a); this difference was significant (t-test, *P* = 0.0018). Among the parthenogens, allelic richness was the lowest in *D. rostombekowi* (16.34) and the highest in *D. uzzelli* (24.33). In all parthenogenic lineages, observed heterozygosity was higher than expected under Hardy-Weinberg equilibrium in a sexually breeding population with similar allele frequencies. These results are consistent with the assumption of their hybrid origins: *D. uzzelli* had the smallest excess of observed heterozygosity, and *D. unisexualis* had the highest.

In sexually breeding species, some deficit of heterozygotes was observed, consistent with the expectation of the Wahlund effect [69] among geographically isolated populations (Fig. 3b). The unexpected result was that all parthenogenetic forms had relatively high proportions of homozygous loci. The highest proportion of homozygous loci was found in parthenogenetic *D. rostombekowi* (60%); while in *D. bendimahiensis* and *D. sapphirina,* the proportion of homozygous loci exceeded 50% (Fig. 3c); Table S1.

### Bayesian inference: clustering of the parthenogens with sexually breeding species

**Fig. 4:**
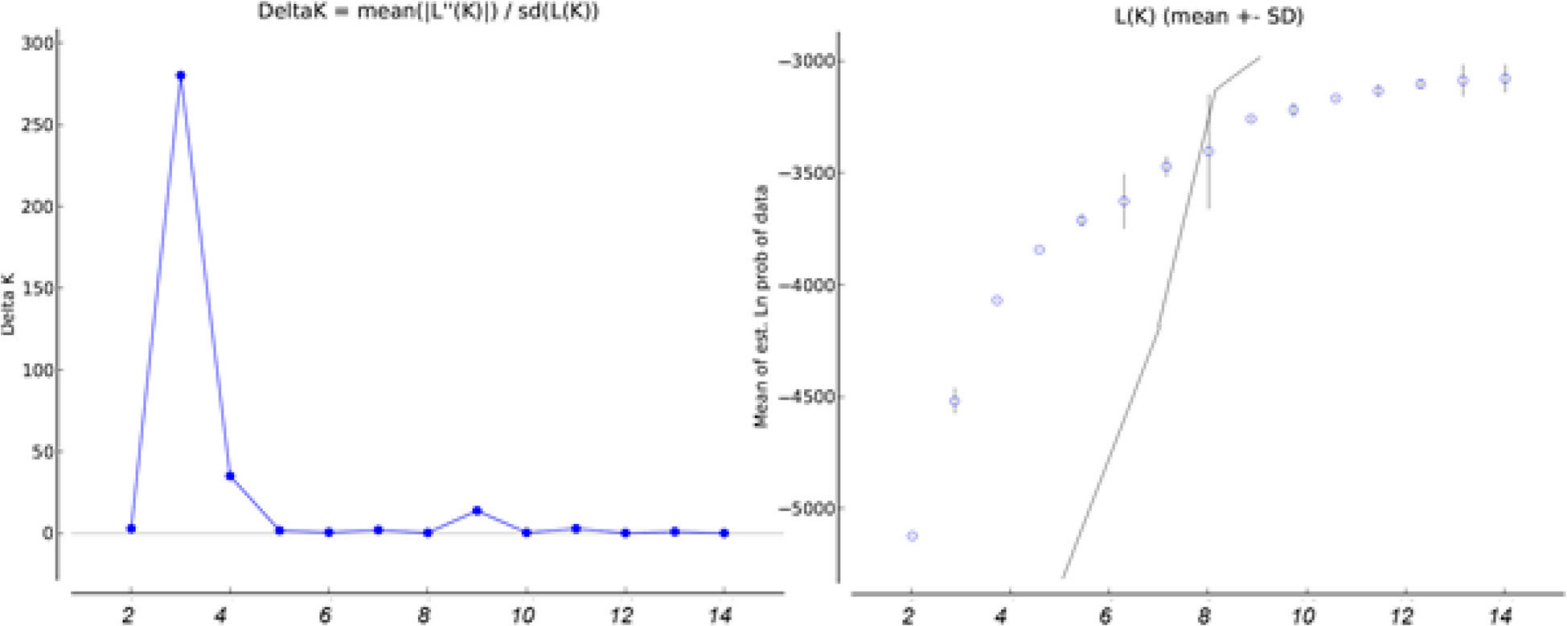
ΔK and BSRK (the number of K selected with broken stick method) based on the clustering of bisexual species only (*D. mixta, D. raddei, D. portschinskii + D. valentini*) with admixture model, 1 = <K = < 15, 10 runs for each analysis

**Fig. 5:**
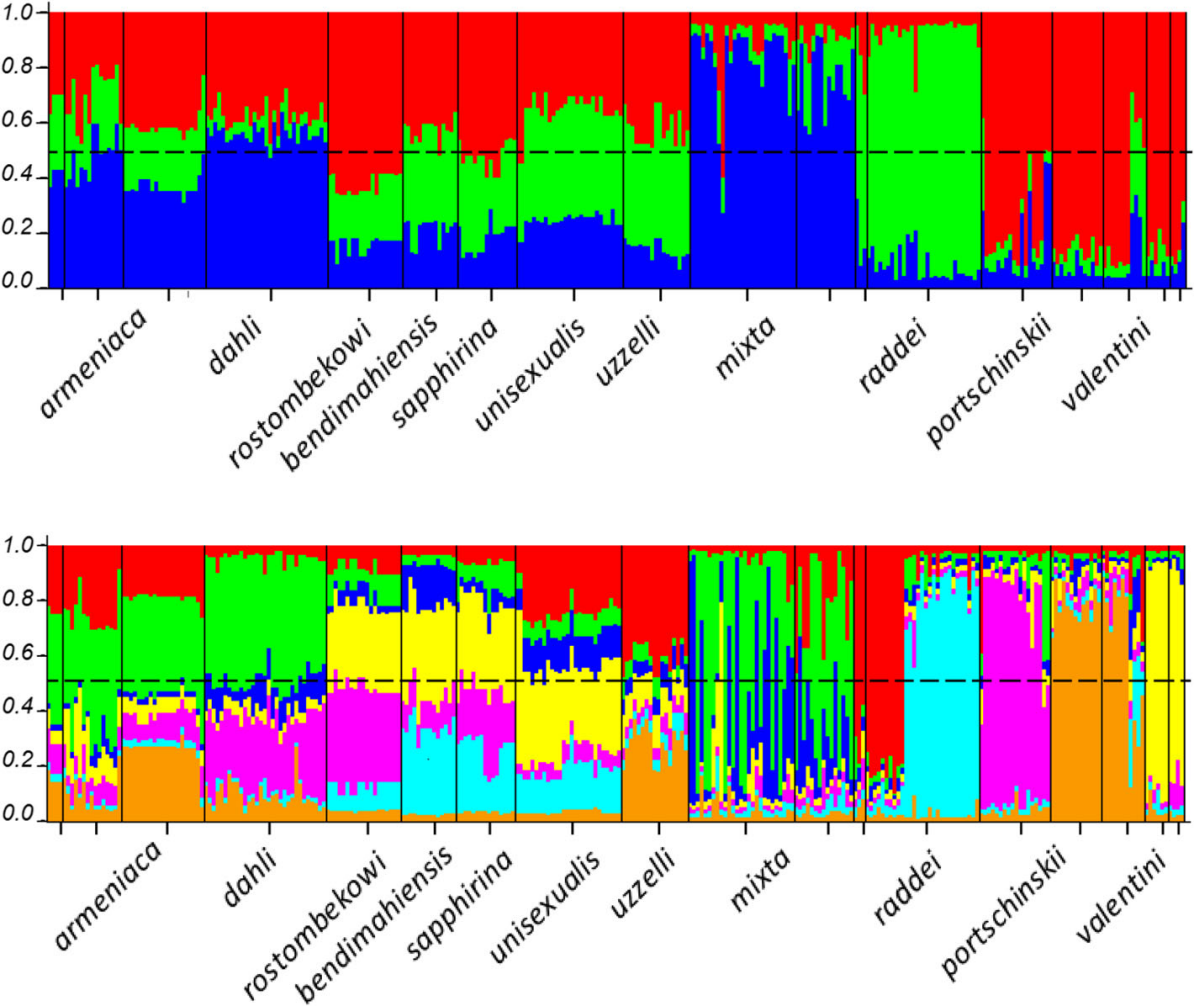
STRUCTURE clustering outcome with K = 3 (upper panel) and K = 7 (lower panel). Admixture model applied, no LOCPRIOR used, POPFLAF = 1 for sexually breeding species (*D. mixta, D. raddei, D. portschinskii, D. valentini*). With K = 7, two geographic populations of *D. mixta* are from the Lesser and the Greater Caucasus respectively; the red cluster marks populations of *D. raddei* from Armenia and northern Turkey, blue cluster - *D. raddei vanensis*, brown cluster - *D. valentini* from Georgia and northeastern Turkey, yellow cluster - *D. valentini* from the Lake Van area

For sexually reproducing species, the highest ΔK was observed at K = 3, while the BSRK method showed the highest support for K = 7 (Fig. 4). Clustering sexually breeding individuals at K = 3 separated *D. mixta, D. raddei,* and a third group comprised of *D. portschinskii* and *D. valentini* individuals from different geographic populations*.* At K = 7, the clustering procedure separated (1, 2) *D. mixta*; *D. raddei* from (3) Armenia and Kars area, and (4) the Lake Van area; (5) *D. portschinskii*; *D. valentini* from (6) southern Georgia and Ardahan area and (7) from the Lake Van area. The separation of the clusters was imperfect, probably due to the presence of ancestral polymorphisms and / or convergent STR alleles (Fig. 5).

At K = 3, we estimated different proportions of each of the three clusters in each parthenogenetic form. The two most common clusters in *D. armeniaca* and *D.dahli* were associated with *D. valentini* + *D. portschinskii* and *D. mixta*. A small proportion of *D. armeniaca* ancestry was attributed to *D. raddei* (Fig. 5).

At K = 7, *D. dahli* and *D. rostombekowi* shared 30–40% of ancestry with *D. portschinskii*, but the latter also had a substantial proportion of ancestry shared with *D. valentini* from the Lake Van region. The latter geographic population also contributed to *D. sapphirina, D. bendimahiensis,* and *D. unisexualis*, whereas *D. armeniaca* and *D. uzzelli* had more shared ancestry with *D. valentini* from southern Georgia and neighboring parts of Turkey (Ardahan). *D. sapphirina, D. bendimahiensis,* and *D. unisexualis* shared most of their ancestry with *D. raddei vanensis,* whereas *D. armeniaca* and *D. uzzelli* appeared to be more associated with *D. raddei nairensis* and *D. r. raddei* (the two latter groups were not distinguishable at K = 7).

All parthenogenetic forms had the most considerable ancestry fraction shared with their presumed parental species, but also possessed a significant contribution from genetic variation associated with other *Darevskia* from the same geographic area (Fig. 5).

### Non-model-based analysis of microsatellite genotypes in parthenogens

#### Genotype diversity

As would be expected in the absence of sexual reproduction in parthenogenetic populations, in each unisexual species we identified 1–3 common genotypes, and a larger number of rare genotypes, that only differed by a few alleles at the individual microsatellite loci (Table 1). The highest diversity of genotypes was observed in *D. armeniaca* and the lowest in *D. sapphirina*. The frequency of the single most frequent genotype varied between 0.207 in *D. dahli* and 0.533 in *D. sapphirina;* the cumulative proportion of the two most common genotypes varied between 0.414 in *D. dahli* and 0.800 in *D. sapphirina.*

**Table 1:** The number of the microsatellite genotypes and their distribution per individuals in seven parthenogenetic species of *Darevskia.* N - Sample size, GT - the number of distinct microsatellite genotypes (all differences at least at one locus considered (individuals with missing data at some loci are treated as distinct genotypes), DR - the Bruvo distance between the two most distant genotypes, MD - the Bruvo distance between the two most common genotypes, 1nd, 2rd, 3rd, 4th - proportion of the first, second, third, and fourth most common genotypes in the sample

	N	GT	DR	MD	1st	2nd	3rd	4th
*D. armeniaca*	44	22	0.266	0.097	0.227	0.091	0.068	0.068
*D. dahli*	29	26	0.594	0.075	0.103	0.069	0.069	0.069
*D. unisexualis*	27	12	0.321	0.153	0.185	0.148	0.148	0.111
*D. uzzelli*	17	12	0.409	0.055	0.176	0.118	0.118	0.118
*D. rostombekowi*	19	5	0.113	0.113	0.474	0.263	0.158	0.053
*D. bendimahiensis*	14	9	0.181	0.028	0.357	0.143	0.071	0.071
*D. sapphirina*	15	7	0.278	0.266	0.267	0.200	0.200	0.133

**Fig. 6:**
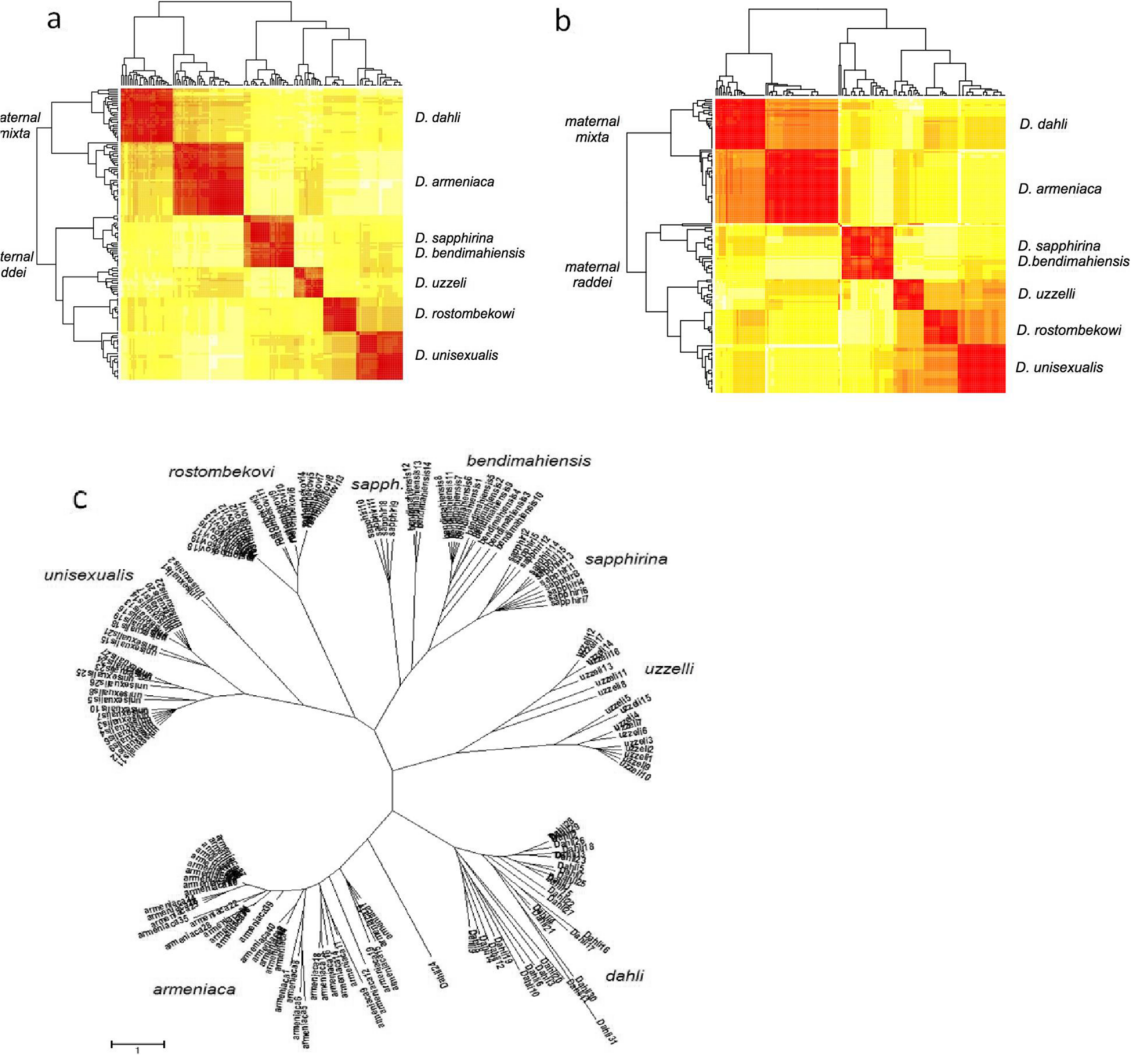
Clustering of all parthenogenetic individuals based on **a**) Pairwise Bruvo distance between microsatellite genotypes represented as a heatmap with NJ cladogram. **b**) Same as in A, but all individuals with no or one heterozygous locus removed in an attempt to remove a possible effect of allele conversion. **c**) An NJ tree was built solely on the pairwise counts of loci with complete shared diploid genotypes (distance between genotypes measured as 10 - no. of shared loci). The colors in the “heatmaps” indicate the distance between the individuals: red color corresponds to small and yellow color to a larger distance

The minimum spanning networks, based on averaged pairwise Bruvo distances between individual microsatellite genotypes, are shown on the NJ cladogram in Fig. 6a and b. There are two main clusters, corresponding to the groups matrilineally descended from *D. raddei* and from *D. mixta*. Within the *D. raddei*-derived cluster, the species with common matrilinear ancestry from *D. r. vanensis* (*D. bendimahiensis* and *D. sapphirina*) formed a tight group with the individual distances between the two species comparable to those within *D. unisexualis*. At the same time, the two parthenogenetic species with mitochondrial ancestry stemming from *D. r. nairensis* (*D. unisexualis* and *D. uzzelli*) did not form a single cluster. Instead, *D. unisexualis* appeared to be much closer to *D. rostombekowi* than to *D. uzzelli* (Fig. 6c).

#### Shared alleles and genotypes between the parthenogens and their presumed ancestral species

Each parthenogenetic species shared at least a few alleles with each bisexual species. *D. dahli* and *D. armeniaca* shared more alleles with their matrilineal ancestor, *D. mixta*, while the other parthenogens had closer links to *D. raddei*. The shared allele pattern on the patrilineal side was less specific, combining alleles found in both presumed paternal populations (*D. portschinskii* and *D. valentini*). When single and two-locus shared diploid genotypes were considered, the similarities between (i) *D. portschinskii* and *D. rostombekowi* (ii) *D. valentini* and *D. bendimahiensis* and (iii) *D. valentini and D. rostombekowi* became more prominent relative to other bisexual-parthenogen pairs.

**Fig. 7:**
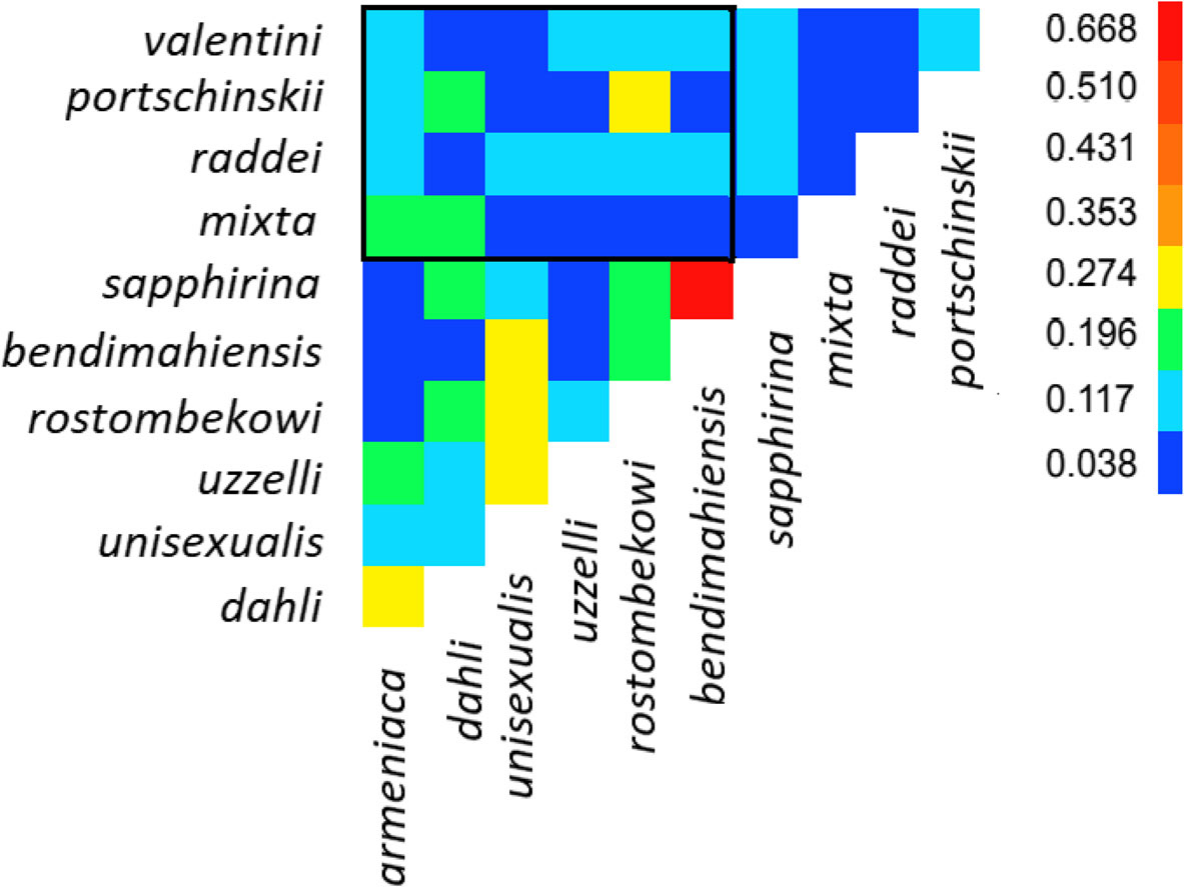
The proportion of shared alleles among the parthenogenetic forms and their presumed ancestors. The colors in the “heatmap” indicate the proportion of shared alleles. Areas outside the black rectangle show the proportion of shared alleles between different parthenogens and different bisexual species, and the area within the rectangle – the alleles of the parental species shared with the parthenogens

Many higher numbers of shared microsatellite alleles and genotypes were observed among the parthenogenetic species. The highest proportion of shared alleles (0.62) was between *D. sapphirina* and *D. bendimahiensis*; followed by *D. dahli* and *D. armeniaca* (0.33), *D. unisexualis* and *D. rostombekowi* (0.34), *D. unisexualis* and *D. uzzelli* (0.29), *D. unisexualis* and *D. bendimahiensis* (0.28), and finally, *D. armeniaca* and *D. uzzelli* (0.24). For single- and two-locus genotypes, the strongest overlap was between (i) *D. bendimahiensis* and *D. sapphirina* (0.49 at single loci and 0.23 at two loci) and (ii) *D. armeniaca* and *D. dahli* (0.30 and 0.07). Substantial overlap was also observed in the following species pairs: *D. rostombekowi - D. bendimahiensis* (0.19 and 0.02), *D. rostombekowi - D. sapphirina* (0.18 and 0.017)*, D. rostombekowi - D. unisexualis* (0.18 and 0.019)*, D. unisexualis - D. bendimahiensis* (0.12 and 0), *D. uzzelli - D. armeniaca* (0.11 and 0.004). The proportion of alleles shared between parthenogens, and their presumed ancestors is shown in Fig. 7.

### Shared single and multilocus genotypes among the parthenogenetic populations: more details

We found that a few specific genotypes were overrepresented in certain pairs of species (Table 2, for complete information on individuals with shared genotypes, see Table S1). The majority of individuals in the *D. armeniaca* - *D. dahli* comparison shared a three-locus genotype, and two individuals also shared a five-locus genotype (Table S1). In *D. bendimahiensis* - *D. sapphirina* pair, the five-locus genotype was shared by most individuals; at the same time, 7 and 3 individuals, respectively, possessed an identical genotype at six loci. Fewer loci (up to 3 in *D. rostombekowi* and *D. unisexualis*) were shared in the same genotype among other pairs of species (Table 2).

**Table 2:** Pairwise comparisons of the most frequent multi- and single-locus genotypes between different parthenogenetic forms of *Darevskia*. Heterozygous genotypes are highlighted in bold

species pair / trio	loci in genotype	alleles in genotype	frequency first species	frequency second species	Prob. of random coincidence ^a^
*armeniaca - dahli*	Du183	**189–193**	0.677	0.875	4.6⨯10^−6^
	Du47	**278–286**			
	Du323	**185–213**			
*bendimahiensis-sapphirina*	Du161	391–391	0.5	0.733	1.83⨯10^−7^
	Du183	**193–201**			
	Du47	282–282			
	Du323	217–217			
	Du215	**204–220**			
*armeniaca-uzzelli (1)*	Du255	204–204	0.5	0.176	1.71⨯10^−3^
	Du215	192–192			
*armeniaca-uzzelli (2)*	Du255	204–204	0.55	0.118	5.73⨯10^−4^
	Du418	140–140			
*unisexualis-rostombekowi*	Du161	395–395	0.074	0.263	4⨯10^−7^
	Du418	136–136			
	Du47	282–282			
*unisexualis-rostombekowi*	Du161	391–391	0.815	0.737	3.03⨯10^−4^
	Du418	136–136			
frequent genotypes shared between three species					
*sapphirina-rostombekowi-bendimahiensis*	Du47	282–282	fixed in all three species		4.14 × 10^−3^
*armeniaca-unisexualis-rostombekowi*	Du418	136–136	0.138; 1; 1		3.28 ⨯10^−4^
*dahli-uzzelli-sapphirina*	Du161	399–399	0.680; 0.353; 0.267		1.17 × 10^−3^
*dahli-uzzelli-bendimahiensis*	Du418	144–144	0.267; 0.667; 0.625		1.79 × 10^−2^

^a^ Probability of random coincidence between two or three parthenogens is calculated on the assumption of random union of the respective parental genotypes during hybridization using current allele frequencies from presumed parental spp

**Fig. 8:**
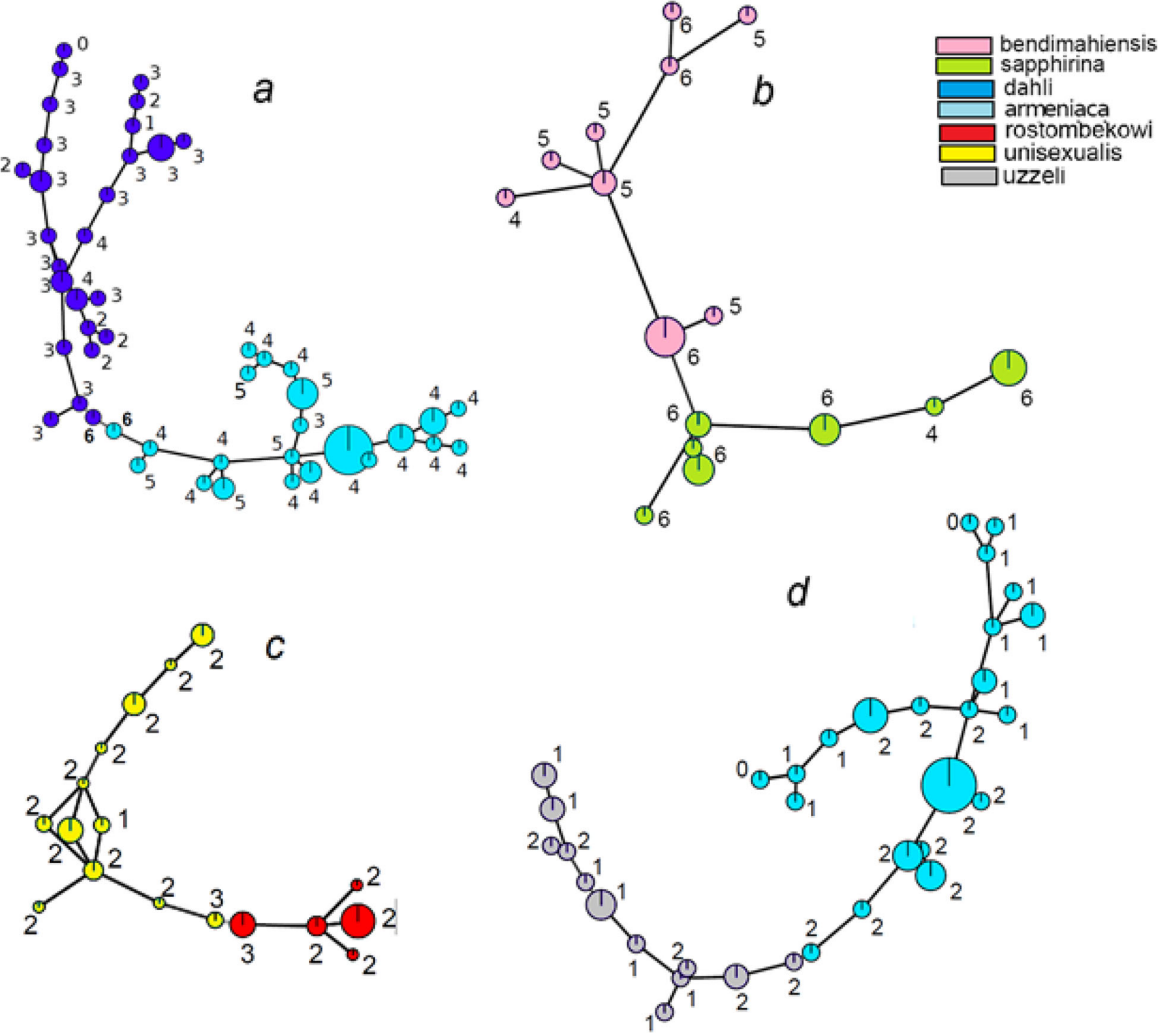
Connectivity of clonal lineages between (**a**) *D. armeniaca* and *D.dahli*, (**b**) *D. bendimahiensis* and *D. sapphirina*, (**c**) *D. unisexualis* and *D. rostombekowi*, (**d**) *D. uzzelli* and *D. armeniaca*, illustrated as Minimum Spanning Networks (MSNs). Each node represents a unique multilocus genotype, with size proportional to the number of individuals. The edges were constructed using Bruvo distances between microsatellite genotypes. The numbers next to the nodes show the maximum number of loci shared with the other species in a pair

To illustrate this pattern in more detail, we overlaid the number of shared loci on the minimum spanning networks based on Bruvo distances between the full multilocus genotypes, in four pairs of the parthenogenetic species with the highest proportion of identical genotypes (Fig. 8). In all pairs, the closest individuals that belong to two different parthenogenetic species also have the maximum number of shared loci (six in *D. armeniaca - D. dahli* and *D. bendimahiensis - D. sapphirina* pair, 3 in *D. rostombekowi- D. unisexualis,* and 2 in *D. armeniaca - D. uzzelli* pair).

The distribution of homozygotes versus heterozygotes among the shared genotypes was also non-random concerning species pairs. That is, *D. armeniaca* and *D. dahli* shared exclusively heterozygous genotypes (Table 2), and a substantial proportion of identical heterozygous genotypes was also observed between *D. bendimahiensis* and *D. sapphirina*. In all other species pairs (and trios), only homozygous genotypes were shared.

**Fig. 9:**
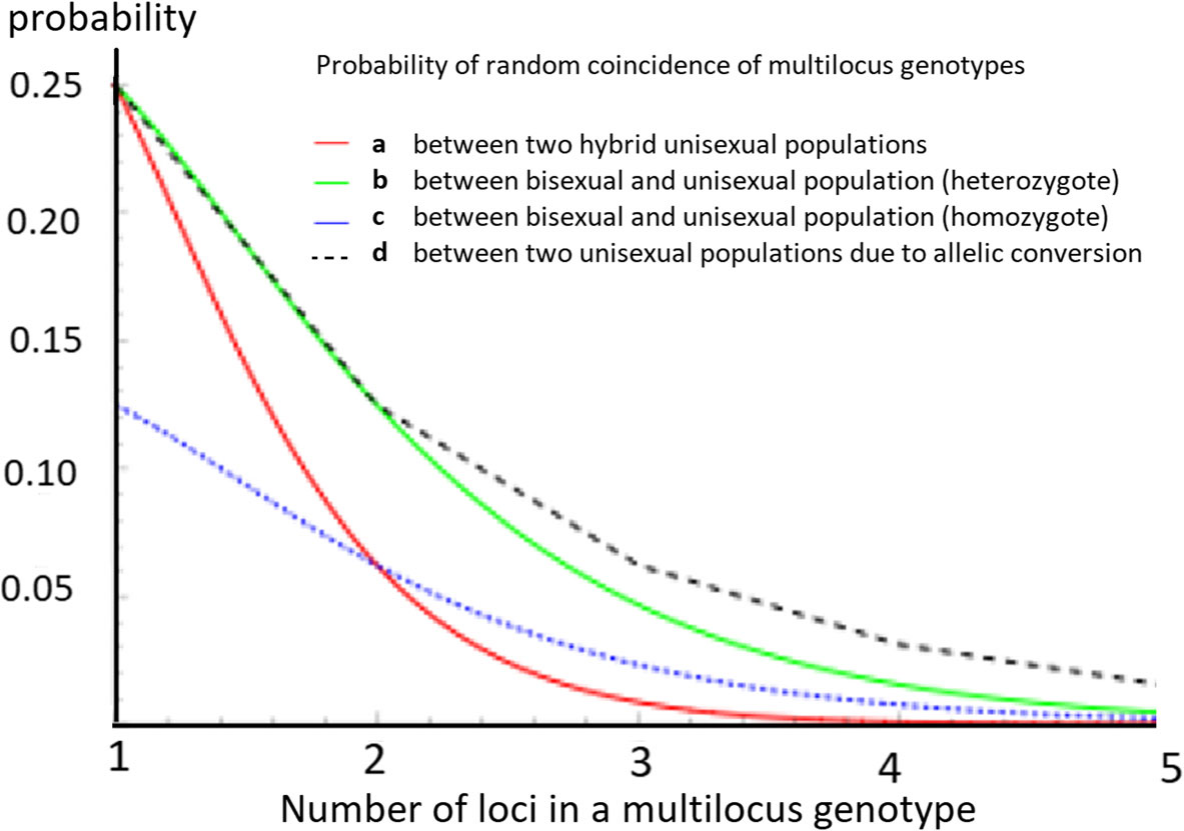
Probability of independent coincidence of a multilocus genotype between (**a**) random coincidence in two unisexual populations (heterozygous *or* homozygous genotypes); (**b**) random coincidence in a parthenogenetic and a bisexual population: all-heterozygous genotype; (**c**) random coincidence in a parthenogenetic and a bisexual population: all-homozygous genotype; (**d**) probability of coincidence in two parthenogenetic populations due to due to allelic conversion (only possible for homozygous genotypes) (see text). X-axis: the number of loci in a multilocus genotype; Y-axis: the respective probability values. All allele frequencies in all populations are set to ½

To verify the null hypothesis that the identical genotypes shared between different parthenogenetic forms may have resulted from n independent coincidence of parental alleles during hybridization of their respective bisexual ancestors, we calculated the theoretical probabilities of independently repeated formation of the individual multilocus genotypes (Table 2, see the Methods for the quantitative approach chosen). A more conservative approach was used to compute homozygous genotypes’ probabilities, assuming they are the result of heterozygosity loss or other forms of gene conversion [28, 70, 71]. Overall, the calculated probabilities were tiny, e.g., in case of the most frequent three-locus genotype shared by *D. armeniaca* and *D. dahli*, *Pad2* = 4.6*10–6. Similarly, the chance of coincidence of a five-locus genotype most commonly shared between *D. sapphirina* and *D. bendimahiensis* is *Psb2* = 1.83*10–7. The probabilities of independent formation of two coinciding two-locus genotypes clustering together most *D. armeniaca* and some of *D. uzzelli* (Table 2), even considering the conservative approach based on the homozygosity of the shared genotype, are 1.71*10–3 and 5.73*10–4, respectively. In other words, if the first species emerged independently as a result of hybridization between a male of *rudis* clade with a female of *D. mixta*, and the second species resulted from hybridization between a male of *rudis* clade with a female of *D. raddei*, in only 1/100 of successful hybridizations would one expect coincidence at the locus Du255, which, coincidentally, is present in over 75% of the individuals of either species. As a purely theoretical exercise, we illustrate the dynamics of coincidence probabilities with respect to the number of loci in a genotype, for hetero- and homozygotes (Fig. 9). Note that all parental allele frequencies in this figure are set to 0.5, i.e., substantially higher than the observed frequency of *any* allele in any presumed parental species, according to our data.

Finally, we show in Fig. 6c that the overall distribution of shared genotypes among parthenogenetic species, as an NJ cladogram based solely on the number of shared genotypes, is almost identical to a direct measure of genetic distance (such as Bruvo distance on full genotypes, Fig. 6a). Both cladograms differ from the mtDNA tree in Fig. 2, showing a closer affinity of *D. unisexualis* to *D. rostombekowi*, rather than to *D. uzzelli*. Note that *D. unisexualis* and *D. rostombekowi* share a high proportion of homozygous genotypes, which could have been caused by the loss of heterozygosity in the clonal lineages. Such a process can inflate genotypic similarities between species and, hence, contribute to the discrepancies between the mtDNA and microsatellite data in this particular case. However, when we excluded all homozygotes from the entire parthenogen dataset and redraw the heatmap, the NJ cladogram’s topology does change (Fig. 6c). The heterozygotes-only heatmap in Fig. 6b demonstrates the presence of three major groups with distinct matrilineal ancestry: (i) *D. mixta*, (ii) *D. raddei vanensis*, and (iii) *D. r. raddei* + *D. r. nairensis.*

## Discussion

Discussion of the origins of parthenogenetic *Darevskia* usually posits several background premises: (1) each of seven parthenogenetic forms of *Darevskia* derives from independent hybridization events [32, 33, 54, 55]; (2) the parental species of the extant parthenogens belong to different clades within *Darevskia* [36, 37, 45]; (3) high heterozygosity of the parthenogens derives from their hybrid origin; (4) all extant parthenogenetic lineages originated in relatively recent geological past, i.e. within the last 200,000 years [32, 36]; (5) genetic exchange between parthenogenetic lineages and sexually reproducing *Darevskia* is rare or nonexistent [32, 37, 61, 72]. Hence, all parthenogenetic lineages of *Darevskia* are expected to be evolutionary dead ends, which, due to high heterozygosity levels ab initio, nevertheless may be successful for short periods [33, 60, 73, 74], and thus provide good examples of geographic parthenogenesis [27, 75].

The analysis presented here suggests that the origin of the parthenogens and their evolutionary perspective is more complicated than previously thought. Parthenogens descend from at least four maternal lineages, and thus four hybridization events. Even more intriguing is that some single- and multilocus microsatellite genotypes are shared between different parthenogenetic forms. Finally, multiple homozygous loci are present in some parthenogens, which is unexpected given their hybrid origin.

### The putative parental lineages of the parthenogens: mitochondrial lineages and recombinant genotypes

Our results confirm and further elaborate previous suggestions [37, 45] on the parental lineages of parthenogenetic *Darevskia*. Mitochondrial DNA sequencing shows that seven parthenogenetic forms of *Darevskia* matrilineally descend from at least four geographic populations. *D. armeniaca* and *D. dahli* descend from *D. mixta* from Borjomi Gorge (see also [24, 67], *D. unisexualis* and *D. uzzelli* descend from *D. raddei nairensis* (northwestern Armenia and the vicinity of Kars in Turkey; see also [36]), *D. rostombekowi* from *D. raddei raddei* (east and south of Armenia), and *D. bendimahiensis + D. sapphirina* from *D. raddei vanensis*. This finding is consistent with the distribution of the presumed maternal cluster component among the same species in our STRUCTURE analysis.

**Fig. 10:**
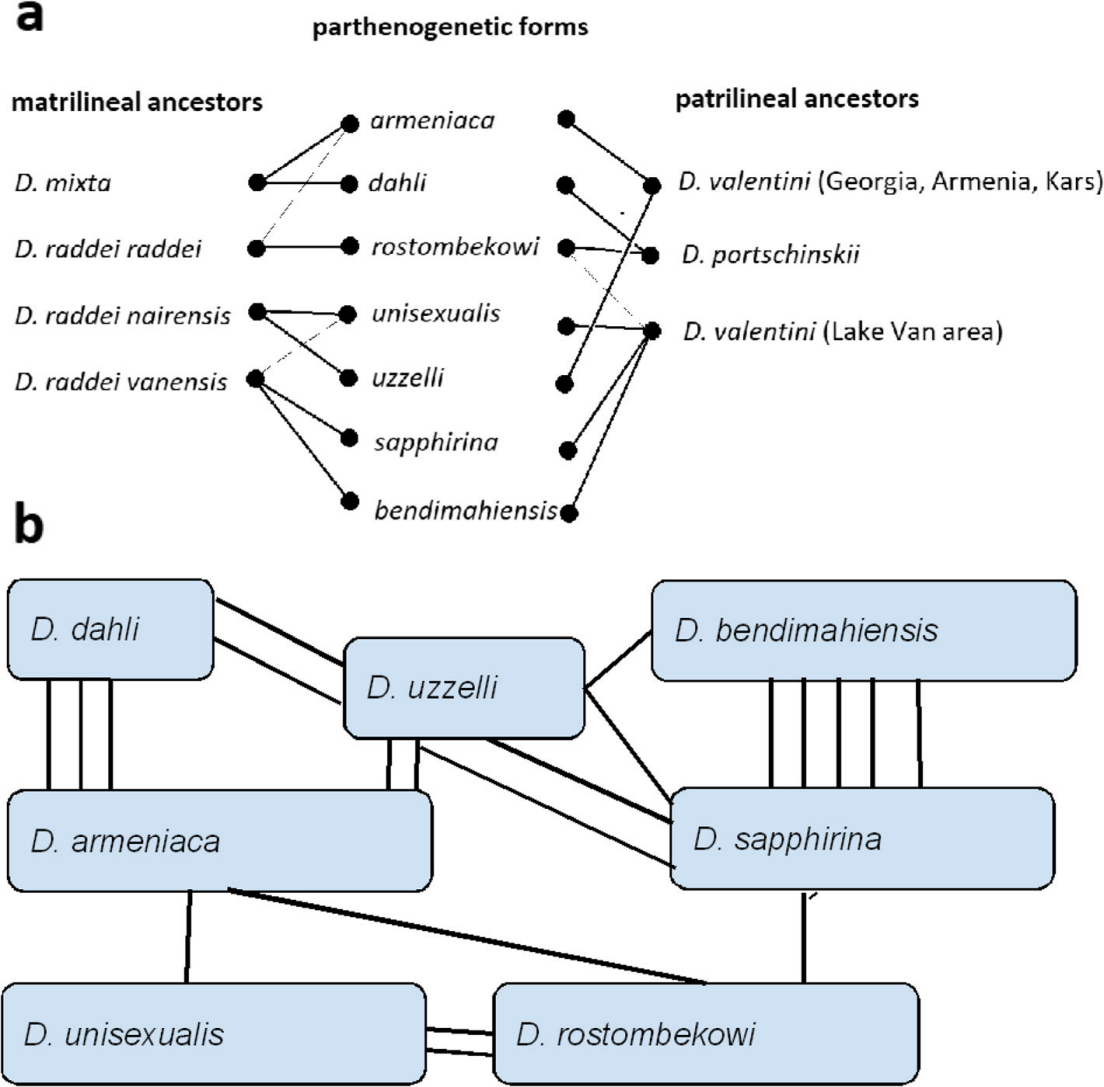
Origin and genotypic overlap in parthenogenetic *Darevskia.* **a** Matrilineal and patrilineal ancestry of seven parthenogenetic forms of *Darevskia*, based on mitochondrial DNA sequences and STRUCTURE analysis at K = 7. Solid lines show matrilineal (left) and patrilineal (right) ancestors of the parthenogens, according to [45] and our data (this paper), specifying geographic populations of the presumed ancestors. Some parthenogens, however, were associated with more than two populations, and dashed lines show the links with these “third” populations (see the text below). **b** The scheme of the most frequent shared genotypes linking the parthenogenetic species of *Darevskia* to each other. Multiple lines demonstrate the numbers of loci with the shared genotypes of different parthenogenetic species

However, patrilineal ancestry remains unclear. *D. portschinskii* and *D. valentini* show a broad introgressive pattern between each other and the neighboring populations of closely related *D. rudis* [46]; hence genetic distances between geographically distant conspecific populations may be greater than between nominally different species from neighboring locations. Therefore, the STRUCTURE results of this group should be interpreted carefully. Our analysis separates (i) *D. portschinskii,* (ii) *D. valentini* from southern Georgia, Armenia, and Kars area in Turkey, and (iii) *D. valentini* from the Lake Van area; the latter is more different from the former two taxa (i-ii) than they were from each other. *D. dahli* and *D. rostombekowi* showed the highest proportion of *D. portschinskii* ancestry; *D. armeniaca* and *D. uzzelli* were most related to the northern *D. valentini* (ii), whereas *D. unisexualis*, *D. bendimahiensis*, and *D. sapphirina* – are more similar to *D. valentini* from the Lake Van area (iii). Finally, *D. unisexualis* shares ancestry between *D. raddei nairensis* and *D. raddei vanensis* in our analysis, consistent with the conclusions of Freitas et al. [37], who used a different set of microsatellite markers; see Fig. 6 in their paper). Figure 10a shows the presumed matrilineal and patrilineal origin of the parthenogenetic *Darevskia,* summarizing the information reported in our paper and previous publications. These findings are only partly consistent with previous analyses that explored the origins of parthenogenetic *Darevskia* [36, 37, 45] and which left open the question, whether more than two ancestral species have contributed to the ancestry of particular parthenogenetic taxa. We also note that occasional similarity in the composition of microsatellite alleles between different presumed ancestral populations might be homoplasy typical in the evolution of microsatellite loci [76].

### Coincidence of microsatellite genotypes between D. armeniaca and *D. dahli*

The case of these two parthenogenetic species is especially puzzling. Murphy et al. [45] hypothesized that *D. armeniaca* and *D. dahli* might have different patrilineal ancestors, based on analysis of allozyme alleles. Matrilineally, both forms undoubtedly derive from *D. mixta*, which could have hybridized with both *D. portschinskii* and *D. valentini* in the past. *D. armeniaca* and *D. dahli* are phenotypically distinct and easy to identify. Adults have different body sizes, coloration, and scalation patterns in the temporal and anal regions [24, 35, 40]. These phenotypic differences are comparable with those between sexually reproducing *Darevskia* species, e.g., *D. portschinskii* and *D. valentini* (the present-day ranges of these two presumed paternal species overlap with *D. dahli* and *D. armeniaca*). More importantly, *D. armeniaca* and *D. dahli* occupy distinct geographic areas and altitudinal ranges (1500–3000 and 800–2000 m a.s.l., respectively), although sometimes individual *D. dahli* are found in marginal locations of *D. armeniaca*. Could these two species emerge from completely different hybridization events?

Tarkhnishvili et al. [24] found two heterozygous single-locus genotypes (at Du47 and Du323) shared at high frequencies in both *D. armeniaca* and *D. dahli*. Vergun et al. [54] and Girnyk et al. [55] genotyped 111 specimens of *D. dahli* and 111 *D. armeniaca* from Armenia using four out of the ten loci included in our analysis (Du215, Du281, Du323, and Du47). Comparing the data between these two studies, the heterozygous genotype comprised of alleles 184 and 211 at the locus Du323 was shared by nearly all specimens of *D. armeniaca* (0.94) and one-third of *D. dahli* (0.27). We have now determined that the vast majority of both species share an identical genotype at three loci, a few also share four- and five-locus genotypes and two individuals even share a six-locus genotype. Calculated from the present-day frequencies of the respective alleles in the parental bisexual species, the probability of accidental fixation of a three-locus *heterozygous* genotype in two individuals descending from different hybrid ancestors is close to zero (Table 2). Of course, if the same combinations of alleles were present at (much) higher frequencies in the actual ancestral populations at the time of initial hybridization, this would increase the chance of coincidence. Although we will never have access to the “true” ancestral populations, the concerted sweep of the same alleles at multiple independent loci from near fixation in the past to very low frequencies at present is just as unlikely. The latter drives us to conclude that a single common hybrid ancestor must have existed for both *D. dahli* and *D. armeniaca*, with a subsequent divergence of the two forms, leading to pronounced phenotypic, ecological, and genetic differences. We further suggest that a backcrossing hypothesis could explain the current genetic differentiation of these parthenogenetic species: i.e., enrichment of gene pools of at least one of the two parthenogenetic forms as a result of backcrossing with one of the parental species [24].

Additional evidence contradicting the hypothetical origin of *D. armeniaca* from direct hybridization between *D. mixta* and *D. valentini* is the structure of ranges of these two presumed parental species, which are separated by a distance of over 50 km (in contrast with *D. mixta* and *D. portschinskii*, the presumed ancestors of *D. dahli*, whose ranges are parapatric).

Hence, the earlier hypothesis of Tarkhnishvili et al. [24] is supported by evidence from additional microsatellite loci and larger samples of the scored loci and individuals. If *D. dahli* is descended from a single hybridization event between female *D. mixta* and a *D. portschinskii* male, *D. armeniaca* could emerge as a result of enrichment of the original parthenogenetic genome by backcrossing with bisexual males. This conclusion is consistent with the results of Freitas et al. [37], which showed an admixture of alleles associated with at least three different presumed ancestral populations, similar to the results published here, but based using different loci. Freitas et al. [37] use the closeness of the proportion of maternal ancestry to 0.5 as an argument for the absence of backcrosses. Our results, however, suggest that the proportion of presumed patrilineal ancestry is significantly different from 0.5. Conclusive validation of this hypothesis will require the analysis of genomic data from the parthenogens and their ancestors.

### Shared genotypes in D. bendimahiensis and D. sapphirina

Two other parthenogenetic forms, *D. bendimahiensis* and *D. sapphirina*, share the highest proportion of identical genotypes (5-locus genotype in most individuals) among all other pairs. Such a coincidence is virtually impossible if the two forms descend from two different hybridization events. Our results show there is very little genetic difference between *D. bendimahiensis* and *D. sapphirina,* those that exist are best explained by the accumulation of mutations in geographically isolated populations that belong to the same hybrid lineage.

### Shared genotypes in other parthenogenetic forms

Figure 10b indicates the presence of shared genotypes in other pairs of the parthenogenetic species, based on the total number of shared genotypes: *D. armeniaca - D. uzzelli* (genotypes shared at three loci), *D. rostombekovi - D. sapphirina* (2 loci), *D. rostombekovi - D. unisexualis* (1 locus), *D. dahli - D. uzzelli - D. sapphirina* (1 locus), *armeniaca - unisexualis - rostombekowi* (1 locus), etc. All parthenogenetic forms share genotypes at at least one microsatellite locus (Fig. 10b).

Shared genotypes are found between species pairs descending from both the same and the different maternal lineages, such as *D. armeniaca* and *D. uzzelli* or *D. rostombekowi* and *D. sapphirina*. In such genotypes, no more than two loci are shared between two individuals (except for a single 3-locus genotype between *D. unisexualis* and *D. rostombekowi*). However, in such cases, the shared genotypes are rare, and they are always homozygous. Below we consider the possibility of homozygous genotypes being the product of allelic conversion, requiring only one allele to be shared originally at a given locus.

### Backcrosses as possible way for diversification of parthenogenetic forms

Descent from a common ancestral hybrid individual explains the presence of shared genotypes at multiple loci between the parthenogenetic species, in particular *D. dahli* and *D. armeniaca*. However, even those pairs of parthenogens with the highest number of shared genotypes also have some loci with fixed or nearly fixed differences. How can these differences be explained?

Microsatellites are highly mutable [77], and the estimated per-locus mutation rate in *Darevskia* reaches 0.1428 per generation [57]. Potentially, one could explain polymorphism within the parthenogens by the aggregation of de novo mutations. However, additional evidence caused multiple authors to interpret genetic variation within the parthenogens as polyclonality, i.e., descent from different F1 hybrids [48, 49, 51, 52, 54, 55]; but see [53]. In our study, the presence of multiple and fixed differences between *D. armeniaca* and *D. dahli* is probably not solely the result of accumulated mutation; at least such extent of divergent phenotypic evolution is unknown in vertebrate parthenogens. We suggest that these genotypic differences (as well as genotypic differences within the forms previously interpreted as polyclonality) could derive from post-hybridization backcrosses with bisexual species of *Darevskia*.

Directional selection in parthenogens is less effective than in sexually reproducing species because clones cannot generate variant genotypes [8]. Moreover, because deleterious mutations are more common than beneficial mutations and difficult to eliminate (Muller’s Ratchet [12]), obligatory parthenogens are thought to be evolutionary “dead ends.” The process of fitness decline in parthenogenetic lineages is accelerated if crossing over between homologous chromosomes of hybrid parthenogens occurs during gonadal maturation: it causes gradual loss of heterozygosity described in detail in [70]. In parthenogenetic *Aspidoscelis* and *Ambystoma*, the lack of recombination between homologous chromosomes prevents loss of heterozygosity (crossing-over between identical sister chromatids still goes on but has no effect) [70, 78] (however meiosis pattern in Darevskia may be different [79]).

The possibility of occasional provision of genes from sexually reproducing individuals into the genomes of parthenogenetic forms alters the evolutionary prospects for asexual lineages. We argue that this is indeed likely in *D. armeniaca* and *D. dahli*. In some populations of *D. armeniaca* and *D. unisexualis*, coexisting with *D. valentini*, the proportion of phenotypically-identified backcross individuals exceeds 36% of the entire parthenogenic population [78]. These authors describe eleven karyotyped backcrossed *D. unisexualis* x *D. valentini* specimens; 5 were triploid females, three triploid males, two intersexual triploid hybrids, and one tetraploid male [62]; another two tetraploid individuals were reported in Freitas et al. [37]. Difficulty in segregating the triploid set of chromosomes during normal meiosis would present an obvious fertility problem to such hybrids [33, 45, 80–82]. Consequently, early authors hypothesized that all hybrid triploids are sterile [58, 61, 72]. On the other hand, successful all-triploid hybrid unisexual lineages exist among parthenogenetic *Aspidoscelis sp.* [29, 70, 83]. In *Darevskia*, no all-triploid parthenogenetic populations have ever been found. However, Arakelyan et al. [62] showed the presence of mature gonads in some triploid females and males, similar to earlier described for *Cnemidophorus* [84]. Spangenberg et al. [63] showed that defects in chromosome synapsis of triploid hybrid male *D. unisexualis x D. valentini* did not block meiosis, although the 281 spermatozoa studied in their paper all showed some developmental defects. In conclusion, backcrossing of parthenogenetic *Darevskia* with their presumed paternal species is documented, although definitive evidence of fertile F1 is still absent.

In vertebrates, perhaps the best-documented evidence of incorporating parental genomes into asexual lineages can be found in North American salamanders *Ambystoma* [78, 85, 86]. In *Ambystoma*, backcrosses between the diploid bisexual species and triploid parthenogens can result in either (i) elevation of ploidy in the parthenogens, or (ii) replacement of one of their three haploid genomes with a new and different one. The offspring ploidy (3n) in the second scenario is not altered, but their genetic diversity is substantially increased [78]. Similarly, strong evidence exists for backcross hybridization and introgression of parental genetic elements into the genomes of gynogenetic Amazon molly fish (*Poecilia formosa*), although the details of the actual cytogenetic mechanism are less clear [19, 20].

In some fish, triploid females can produce haploid eggs [87]. The chance that a gamete containing a copy of each chromosome is produced following triploid meiosis decreases exponentially with the number of chromosomes. The probability of meiosis in which all univalents pass to the same pole is equal to 0.5^(x-1), where x is the number of haploid chromosomes in gene pool [88]. *Darevskia* have *n* = 19 chromosomes in a haploid complement [32, 63, 89]; hence, the probability of producing haploid or diploid gametes by a triploid individual is 0.0000038147 (one out of 262,144 gametes). This is certainly a rare event; however, considering the relatively high proportion of triploid individuals in some parthenogenetic populations and the high number of gametes produced by males, viable gametes may appear occasionally.

### Homozygous loci in parthenogens

We assume that two allelic copies at each locus are inherited from the maternal and the paternal bisexual parents. This assumption is consistent with higher observed overall levels of heterozygosity in the parthenogens compared to bisexual taxa. However, homozygous loci are common in *D. rostombekowi* (six out of ten scored loci in most individuals), *D. bendimahiensis,* and *D. sapphirina* (up to five loci) and *D. unisexualis* (four loci).

The frequencies of the respective alleles differ strongly between the putative maternal and paternal ancestors, at least in their respective present-day populations. Unless genetic drift in the bisexual populations has been particularly strong since hybridization, the chance of the random generation of a homozygous individual, one who is homozygous at so many loci, is meager. The fact that we observe multilocus homozygous genotypes shared between different parthenogenetic species, (e.g., 3-locus homozygous genotype is shared between *D. unisexualis* and *D.rostombekowi*) is even more puzzling, and requires considering alternative processes that increase the frequency of homozygotes post-hybridization.

Indeed, the loss of heterozygosity in hybrid parthenogenetic lineages is a frequently observed phenomenon [6] and can be caused by pairing and crossing over homologous non-sister chromosomes. The segregation of recombinant gametes is expected to result in a random dropout/meiotic conversion of one or the other allele from the clonal lineage and increased homozygosity of the parthenogenetic population. On the other hand, Lutes et al. [70] described an efficient mechanism of maintaining heterozygosity in hybrid parthenogenetic lizards (*Aspidoscelis*). According to this paper, pairing and crossing-over occur only between the identical sister chromatids, which effectively rescues the unisexual lineage from the rapid loss of heterozygosity. The same process has been observed in mole salamanders [21]. Although unisexual forms of both *Aspidoscelis* and *Darevskia* are seemingly able to maintain heterozygosity, a general mechanism described in Lutes et al. [70] might not always work to perfection: rare occasional recombination between homologs would inflate the frequencies of homozygotes over time. The meiotic mechanism in the diploid parthenogenetic *Darevskia* has not yet been described in sufficient detail; however, Spangenberg et al. [63] observed crossing-over in trivalent of non-sister homologous chromosomes in a triploid male from a cross between *D. unisexualis* × *D. valentini*. This possibility suggests that homologous recombination *is* possible, at least under certain conditions.

Alternative mechanisms of gene conversion are known, but not for vertebrate parthenogens. For instance, in bdelloid rotifers, oocytes are formed through mitotic divisions, with no evidence of chromosomal pairing [90]. In this case, gene conversion can be biased towards one or the other allele, thereby driving the proportions of the parental genomes from 1:1 ratio. Finally, elements of one parental genome may be directly favoured by natural selection in the parthenogens. Interestingly, the proportions of homozygous loci shared between *D. rostombekowi*, *D. dahli*, and *D. bendimahiensis* and, respectively, either of their bisexual parents is visibly biased towards the paternal alleles (*D. portschinsk*ii and *D. valentini*).

As we mentioned before, the proportion of homozygous loci differs between the parthenogenetic forms*.* The loss of heterozygosity is a slow process, a product of genetic drift, hence taking thousands of generations to have an effect. Different proportion of homozygous loci in different parthenogenetic lineages is explained either with different age, or different population size. The parthenogens with the lowest proportion of homozygous loci have broader ranges and consequently probably have larger populations than those with a higher proportion of homozygous loci. Therefore, the time it takes for an allele to reach fixation is directly proportional to the population size of a given parthenogenetic form. The high proportion of homozygous loci in *D. rostombekowi* could reflect its small range and small population size. The loss of heterozygosity inevitably leads to the decline of fitness, and eventual extinction of unisexual lineages [8, 11, 12, 91], *unless* the parthenogenetic genotype is enriched as a result of an extraspecific augmentation of genetic diversity.

### Hypotheses related to parthenogenesis in rock lizards: a synthesis

Here we approach a critical question raised by previous authors [32, 34, 37, 45, 72]: how often does hybridization between different bisexual lineages lead to parthenogenesis? Theory predicts that the number of genetic incompatibilities increases exponentially with the number of nucleotide differences between diverging populations, amounting to ever more substantial barriers to gene exchange [92]. Chromosome pairing and segregation of gametes during meiosis is a complex process, where incompatibilities may cause hybrid infertility or inviability [93]. However, a switch to parthenogenetic reproduction may not require compatibility between homologous parts of the genome. In spiny loaches (*Cobitis*), hybrid parthenogenesis occurs only at an advanced stage of divergence [80]. Darevsky [40] suggested that parthenogens may result from hybridization between *any* species that are diverged insufficiently to prevent hybridization completely, but sufficiently different to undermine the “normal” mechanisms of gametogenesis. By contrast, Moritz et al. [33] argued that only *specific* evolutionary lineages can produce parthenogens upon hybridization, irrespective of the evolutionary distance between them. Neither of these hypotheses is completely validated or disproved so far; and, as we will see, this discussion remains important *only if* all parthenogenetic lineages of rock lizards indeed derived from F1 hybrids between different sexually reproducing individuals.

The results reported here question this assumption. At least in the case of *D. dahli - D. armeniaca*, development of one of the two parthenogenetic lineages could have included backcrosses with the male of the paternal species, whose offspring then switch to exclusively parthenogenetic reproduction.

Interestingly, the ranges of all parthenogenetic *Darevskia* either overlap or are parapatric [36, 40]. They form a single geographic area between the mountains of the Central Lesser Caucasus and Lake Van. Even though representatives of the *rudis* and *caucasica* clades coexist throughout the Greater Caucasus and the Western Lesser Caucasus (Doğu Karadeniz in Turkey), no parthenogenetic forms have ever been recorded there. This fact, together with the presence of overlapping microsatellite genotypes, forces us to challenge the dominant hypothesis about the independent origin of seven parthenogenetic *Darevskia* and search for additional potential explanations.

North American whiptail lizards (*Aspidoscelis*), similar to *Darevskia*, include up to 15 hybridogenous parthenogenetic forms (http://reptile-database.reptarium.cz/). The origin of these parthenogens remains a subject of extensive discussion, comparable with that on *Darevskia*. Five out of seven parthenogenetic *Aspidoscelis* from New Mexico are triploid [94, 95]. The presence of triploid forms is clear evidence of successful backcrosses between the diploid parthenogens and their sexually breeding relatives [96]. However, these backcrosses giving rise to new parthenogenetic lineages have never been observed in nature, suggesting that the phenomenon is unusual. The latter is not surprising for the reasons discussed above for *Darevskia*: the chance to find an instantly reproductively successful triploid hybrid is meager. Cole et al. [97] and Lutes et al. [96] managed to produce a hybrid tetraploid parthenogenetic lineage of *Aspidoscelis* in laboratory conditions, which can be taken as evidence of such a possibility in nature.

## Conclusions

We argue that enrichment of parthenogenetic genetic variation by occasional backcrosses might exist in Caucasian rock lizards, and potentially in other parthenogenetic reptiles. Backcrossed polyploid hybrids are relatively frequent in *Darevskia*, although no direct evidence of recent gene flow has been previously documented; here we showed coincidence of multilocus genotypes in different species of parthenogenetic rock lizards, a fact difficult to explain if gene flow between the parthenogens and their sexually breeding ancestors is excluded. Our results suggest that the mechanism of maintaining heterozygosity described earlier [70] may be imperfect, and the parthenogens are gradually losing heterozygosity as a result of the allelic conversion. Hence, their fitness is expected to decline over time as genetic diversity declines. Backcrossing with the parental species could be a rescue mechanism that might prevent this decline and increase the persistence of unisexual forms.

The apparition of a new hybrid parthenogenetic lineage from sexually reproducing parents is an exceptionally rare event in reptiles—simple coexistence of species from specific lineages or lineages separated at a specific evolutionary distance is an insufficient precondition for such evolutionary novelty. However, a parthenogenetic lineage may survive for a long time and adapt to changing environments if backcrosses occasionally enrich its genome.

## Methods

### Sampling

Samples for DNA analysis were collected between 2010 and 2018 in Georgia, Turkey, and Armenia (Fig. 1). All individuals were hand-caught, temporal, dorsal, and anal areas photographed for verification of species (available upon request, also see Tarkhnishvili et al., 2010, [46] for details), and tail-tips removed from live animals for DNA extraction. Procedures for live-animal handling were approved by the Animal Research Ethics committees of Ilia State and Bülent Ecevit universities. In all instances, the species could be identified unambiguously in the field: in the case of morphologically similar *D. bendimahiensis* and *D. sapphirina*, the samples were collected from the respective *terrae typica* [42]. We collected samples from 19 populations of seven parthenogenetic *Darevskia*, and 14 populations of four presumably ancestral bisexual species (Table 3, Fig. 1). Samples of the parthenogens included 44 D. armeniaca, 29 *D. dahli*, 28 D. unisexualis, 17 D. uzzelli, 14 D. bendimahiensis, 15 D. sapphirina, and 19 D. rostombekowi altogether. Samples of the presumed bisexual ancestors included 42 *D. mixta*, 6 *D. raddei raddei*, 10 *D. raddei nairensis,* 19 *D. raddei vanensis*, 33 *D. valentin*i, and 15 *D. portschinskii*.

**Table 3:** The number of samples and the localities of the seven parthenogenetic *Darevskia* species and their presumed ancestors

Species/ population	Location as shown in Fig. 1	MtDNA (our samples)^a^	MtDNA (GenBank) ^b^	GenBank Accession #	STR profiles^c^
*D. armeniaca*	1 - Hrazdan	2	Armenia - 2	MH247120, AF147799	**4**
	2a,b – Didgori, Khospio	3	Georgia- 46		**17**
	3a,b - Ardahan, Çıldır	0	Turkey-0		**21**
*D. dahli*	4a,b – Kojori, Didgori	2	Georgia – 0 Armenia-1	AF147800	**29**
*D. bendimahiensis*	5a,b – Muradiye, Çaldıran	2	Turkey - 2	MH271047, MH271049, MH271050, AF164084,	**14**
*D. sapphirina*	6a,b – Patnos, Pınarlı	2	Turkey - 1	AF164083	**15**
*D. rostombekowi*	7 - Dilijan	2	Armenia - 3	AF164089-AF164091; MH247116	**19**
*D. unisexualis*	8a – Hrazdan	0	Armenia - 9	AF164085-AF164088 MH271034-MH271035 MH271053-MH271054 MN015183	**2**
	8b,c,d – Hanak, Horasan, Ağrı	3	Turkey - 0		**20**
*D. uzzelli*	9a,b,c- Kars, Sarıkamış, Horasan	2	Turkey - 5	MH271037-MH271039 MH271041, AF164082	**17**
***D. mixta***	10a,b – Akhaldaba, Abastumani	0	Georgia - 10	KM496573-KM496579 AF147796 - AF147798	**25**
	11 - Ambrolauri	0	Georgia - 3	KM496580- KM496582	**17**
***D. raddei*** *raddei*	12a - Sotk	2	5 (Armenia)	MH247117, MH271052, MH271060, MH271063, AF164075	**3**
***D. raddei nairensis***	12b	2	Armenia-16	AF164074, AF164077-AF164081; U88606, U88607, U88613; MH247032, MH247051, MH247055, MH247057, MH247113-MH247116	**10**
	12c - Vardzia	3	Georgia-0		**3**
***D. raddei vanensis***	13a,b – Doğubeyazıt, Muradiye	4	2 (Turkey)	MH271045, AF164076	**19**
***D. raddei***	Northern Iran (undefined)		3 (Iran)	KF717239, KF717241	**0**
***D. portschinskii***	14a,b – Khrami, Kojori	N/A	Georgia	N/A	**10**
	14c – Mets Sapasar	N/A	Armenia	N/A	**3**
***D. valentini***	15 - Akhalkalaki	N/A	Georgia	N/A	**13**
	16 - Ardahan	N/A	Turkey	N/A	**7**
	17 - Erzurum	N/A		N/A	**5**
	18 - Çaldıran	N/A		N/A	**4**

^a^ the number of MtDNA sequences generated and analysed within current research

^b^ MtDNA sequences downloaded from GenBank, used in the present paper; the area of origin reported in GenBank nearest to the respective sampling location in our data is shown

^c^The number of individual microsatellite genotypes generated and analysed in the current paper

### DNA extraction, sequencing, and analysis of mtDNA haplotypes

DNA was extracted from tissue samples using a Qiagen tissue kit (QIAamp DNA Mini Kit, Qiagen, Germany) according to the manufacturer’s instructions [98]. To check for contamination and pipetting error, a negative control (reagents only) was used for each extraction procedure and PCR. Two different primer pairs (H15915 – L15369 and H15488 – L15153, [45, 48]) were used for amplification of a 683 bp fragment of the cytochrome b mitochondrial gene [24, 45, 46]. The PCR conditions were as follows: 20 μl total volume, with 2–4 μl template DNA, 1 U of GoTaq Flexi DNA Polymerase (Promega), 1X GoTaq Green Flexi DNA Polymerase buffer, 1 mM of MgCl2, 0.2 mM of each dNTP and primer concentrations at 0.1 μM. The PCR profile included initial denaturation at 93 °C for 3 min, followed by 30 cycles at 93 °C for 1 min, 53 °C for 1 min, and 69 °C for 2 min and 70 °C for 10 min for the final extension. The amplicons were sequenced on an ABI 3130 sequencer in both directions to ensure sequence accuracy. Sequences were edited using SEQSCAPE 2.5 (Applied Biosystems Inc. Foster City, CA, USA), and unique sequences (haplotypes) were deposited in GenBank (accession no KM496573–82).

We used mitochondrial sequences of *D. mixta* and its daughter parthenogens *D. armeniaca* and *D. dahli*, as well as the sequences of *D. raddei* and its daughter parthenogens *D. unisexualis, D. uzzelli, D. bendimahiensis, D. sapphirina,* and *D. rostombekowi* to identify the most likely maternal population of each parthenogenetic form. Mitochondrial DNA analysis included 46 samples of *D. dahli,* 20 of *D. armeniaca,* and 14 of their presumed matrilineal ancestor *D. mixta* obtained in an earlier study [24], some of these samples were re-analyzed for a longer fragment of the *Cyt-b* gene (683 bp) (Table 3). We included all homologous cytochrome b sequences of parthenogenetic *Darevskia* and their presumed matrilineal ancestors, *D. raddei* and *D. mixta*, available from GenBank, along with the novel sequences obtained for this study. The total number of individuals of each species, the GenBank accession numbers, and their source populations are provided in Table 3.

To infer the most likely local matrilineal ancestor populations of the parthenogenetic species descended from *D. raddei*, we reconstructed the haplotypes tree as well as the haplotype network— Maximum Likelihood (ML) tree of seven parthenogenetic taxa and their presumed ancestors was reconstructed using MEGA 7.0 [99]. The best-fit substitution model and prior specifications for ML analysis were inferred with the same software (Maximum Likelihood method was applied); the Model with the lowest BIC scores (Bayesian Information Criterion) was considered to describe the substitution pattern the best. The support for branching patterns was estimated by 500 bootstrap replications. Considering reciprocal monophyly of *D. mixta* and *D. raddei* [45, 66]*,* we used the former species and its daughter parthenogens as outgroups with respect to *D. raddei* and its daughter parthenogens.

The Median-Joining network [100] was used for illustrating the degree of differences between individual haplotypes of *D. raddei* and haplotypes of parthenogens matrilineally descending from this species (*D. unisexualis, D. uzzelli, D. bendimahiensis, D. sapphirina, D. rostombekowi*). The software NETWORK 5.0 [100] was used. A similar network showing the differences between individual haplotypes of *D. mixta* and its daughter parthenogens *D. armeniaca* and *D. dahli* was published earlier [24].

### Microsatellite genotyping

We inferred genotypes of 289 individual lizards at ten microsatellite loci: Du161, Du183, Du231, Du255, Du365 [101], Du418, Du47, Du281, Du323, and Du215 [102]. However, in 142 individuals, the locus Du418 could not be reliably scored, hence approximately half of the individuals were studied for nine and not ten loci (maximum number of missing loci per individual = 3, the overall proportion of missing data = 12.39%). Microsatellite PCR reactions were performed using the Qiagen Multiplex PCR kit with primer concentrations of 0.11–0.14 μM (ca 0.1 mm). Thermal cycling was performed at 95 °C for 15 min, followed by touchdown of 15 cycles of 94 °C for 30 s, 56 °C for 45 s incorporating a stepwise decrease of 0.5 °C at each cycle, 72 °C for 1 min, then 22 cycles of 94 °C for 1 min, 48 °C for 90 s, and 72 °C for 1 min, followed by a final extension at 60 °C for 30 min. PCR multiplexes were developed to reduce the time and cost of genetic analyses. We amplified the ten loci in three multiplex PCR reactions with following primer combinations: multiplex I - Du161, Du231, Du255, Du183, Du365; multiplex II: Du47, Du281, Du418 and multiplex III: Du323 and Du215 Amplicons were run on a 3130*xl* Genetic Analyser (Applied Biosystems), using deionized formamide and Genescan size standard LIZ 500 (Applied Biosystems Inc. Foster City, CA, USA). We amplified and scored all loci at least three times, and calculated genotyping errors (allelic dropouts, false alleles, and genotype reliability), following the guidelines described in [103, 104]. We set the reliable change index (RCI) threshold value as 95% and verified every individual twice for heterozygosity, and three times for homozygosity, since allelic dropouts are less likely to be detected at homozygous loci [105].

Since we frequently observed identical genotypes shared among the different parthenogens (see the Results section), we first validated the trivial hypothesis that these coincidences resulted from misidentification of genotypes. We examined the cases when alleles in the high-frequency genotypes of the parthenogens were also shared with one or more bisexual species, and tested their frequencies for HW proportions in the respective bisexual populations.

### Analysis of allele and genotype frequencies and differentiation between taxa

We used the R packages *adegenet* [106], *hierfstat* [107], and *PopGenReport* [108] to infer allele and genotype diversity estimates within and between conspecific populations of all parthenogenetic species and their presumed ancestors.

The proportions of shared alleles were calculated per individual pairs of parthenogenetic species using the function propShared in *adegenet* and averaged across the respective groups. The same approach was used when calculating the proportions of shared multilocus genotypes, i.e., by treating each combination of a given number of loci as one haploid locus and each corresponding genotype as an allele. The minimum spanning networks [109], based on averaged pairwise Bruvo distances between individual microsatellite genotypes, were built using the R packages *poppr* [110, 111] and *igraph* [112]. The Bruvo distance [113] between two microsatellite alleles is calculated as:

1 $$ d=1-\left({2}^{-\left|x\right|}\right) $$

where x is a number of repeat units that set the two alleles apart.

### Bayesian analysis of the microsatellite genotypes

STRUCTURE v2.2 [114] separated the dataset into groups with the least within-locus and between-locus disequilibria. Bayesian clustering is a standard method of population analysis, especially in cases where hybridization is involved [114]. However, populations of clonally reproducing organisms do not satisfy the main assumption of STRUCTURE, i.e. that genotype frequencies are independent and tend towards linkage equilibrium. For this reason, we first ran the analysis for bisexual species only, to infer the number of clusters with the highest log-likelihood. The procedure was repeated ten times for each a priori delimited number of clusters (K), ranging from 2 to 15, for each of three settings: (1) admixture model without a priori information on the location (no LOCPRIOR option); (2) no admixture model without LOCPRIOR option applied; (3) no admixture model,

with LOCPRIOR option applied. MCMC parameters were set with a burn-in period of 100,000 and 100,000 post-burn-in replicates. The admixture model without a priori information on the location showed fully consistent clustering among different runs; hence we inferred the population structure based on the setting (1). We calculated ΔK statistics based on the difference between two consequential K values [115, 116] using Structure Harvester website [117]. Since the log likelihood monotonically increased with K, we considered K corresponding to the highest ΔK as the most likely number of ancestral population clusters before the interspecific hybridization/first appearance of the parthenogenetic hybrids. Additionally, we inferred the number of clusters satisfying the broken-stick rule (BSRK [118];). The resulting value of K with the highest support was then used in the subsequent analysis involving both sexual and parthenogenetic populations, following the procedure described in [119].

We next estimated the ancestry proportions gained by each parthenogenetic individual from the anticipated bisexual parental clusters.

We assigned POPFLAG = 1 to sexual species and POPFLAG = 0 to asexuals, which treats parthenogens as admixed individuals of unknown origin. In this analysis, MCMC parameters settings were as in the clustering bisexual species; both for K=K|max (ΔK) and K=BSRK, the procedure was repeated ten times, and the run with the highest Ln P(D) [115] was chosen and discussed.

#### Probability of independent coincidence of multilocus genotypes

In cases where the same multilocus genotypes were identified in two or more parthenogenetic species, we tested the null hypothesis of their independent and random coincidence. Based on the assumption that a specific parthenogenetic genotype had resulted from hybridization between the maternal (*D. mixta* or *D. raddei*) and paternal (*D.valentini* and *D.portschinskii* combined) bisexual taxa, and given the frequencies of the respective alleles in the putative ancestors:

2 $$ {P}_{\left(L,n\right)}=\sum \limits_i^{2n}\prod \limits_{i\ne j}^n{p}_{li}{q}_{lj} $$

where *P*_*(L,n)*_ is the probability of a hybrid multilocus genotype *L* at *n* loci; *p*_*li*_ - frequency of allele *i* at locus *l* in the paternal bisexual population; *q*_*lj*_ - frequency of allele *j* at locus *l* in the maternal bisexual population.

For example, for two loci *A* and *B*:

$$ {P}_{A_1{A}_2{B}_1{B}_2}={p}_{A_1}{p}_{B_1}{q}_{A_2}{q}_{B_2}+{p}_{A_2}{p}_{B_1}{q}_{A_1}{q}_{B_2}+{p}_{A_2}{p}_{B_2}{q}_{A_1}{q}_{B_1}+{p}_{A_1}{p}_{B_2}{q}_{A_2}{q}_{B_1} $$

where ${P}_{A_1{A}_2{B}_1{B}_2}$ is the probability of a hybrid genotype with the alleles *A*_*1*_ *A*_*2*_ and *B*_*1*_*B*_*2*_; *p*_*A1*_ - frequency of *A1* in the paternal population, *q*_*A1*_ - frequency of *A1* in the maternal population, etc. If the genotype in question was homozygous, we applied a conservative approach, considering that the homozygosity could be a result of the allelic conversion, leading to loss of heterozygosity when only one parental allele is retained at a locus (see Results and Discussion). In this case, the probability of a parthenogenetic, homozygous locus with allele *i* was considered equal to a sum of frequencies of this allele in both presumed parental taxa divided by two (assuming that the probability of loss of each of two alleles in a genotype is the same). Hence, we considered

3 $$ {P}_{(Ln)}=\prod \limits_{l=1}^n\left(\frac{p_{li}}{2}+\frac{q{}_{li}}{2}\right) $$

where *p*_*li*_ and *q*_*li*_ are the frequencies of the allele *i* at locus *l* in the presumed parental populations. These frequencies are multiplied with each other and (if applicable) with those calculated using eq. (2). To calculate the probability of random coincidence, i.e. the chance of occurrence of the same hybrid genotype twice, the respective values in (2) or (3) were squared. In case of single locus genotypes that were shared between three different parthenogenetic species, the probability of chance coincidence of the respective genotypes was calculated by raising the respective values of (3) to the third degree.

## Supplementary information

**Additional file 1 **: **Table S1**. Excel file with microsatellite genotypes of all studied parthenogenetic and sexually reproducing individuals, with the indication of species name.

## Data Availability

All data generated or analysed during this study are included in this published article and its supplementary file. All individual STR profiles are presented in supplementary Table S1; GenBank accession numbers of additional unique sequences produced in the framework of current study are given in the text of the paper.

## Abbreviations

BIC: Bayesian information criterion
BSRK: The optimal number of clusters K, based on Bayesian STRUCTURE analysis, selected with broken stick method
K: The number of clusters, studied with Bayesian STRUCTURE analysis
MCMC: Markov Chain Monte Carlo
ML: Maximum likelihood
RCI: Reliable change index
STR: Short tandem repeats

## References

[1] Suomalainen E (1950). Parthenogenesis in animals. Advances in Genetics.

[2] Suomalainen E, Saura A, Lokki J (1987). Cytology and evolution in parthenogenesis.

[3] White MJD (1945). Animal Cytology and Evolution.

[4] White MJD (1984). Chromosomal mechanisms in animal reproduction. Bollettino di zoologia..

[5] Maynard-Smith J (1978). The evolution of sex.

[6] Maynard-Smith J (1989). Evolutionary Genetics.

[7] Bell G (1982). The Masterpiece of Nature: The Evolution and Genetics of Sexuality.

[8] Bell G (2008). Selection: the mechanism of evolution.

[9] Templeton AR, Ashburner M, Carson HL, Thompson JN (1983). Natural and experimental parthenogenesis in Drosophila. The Genetics and Biology of Drosophila.

[10] Bengtsson BO, Schön I, Martens K, Dijk P (2009). Asex and evolution: a very large-scale overview. Lost sex.

[11] Kondrashov AS (1988). Deleterious mutations and the evolution of sexual reproduction. Nature..

[12] Muller HJ (1932). Some genetic aspects of sex. Am Nat..

[13] Doust JL, Doust LL (1988). Plant reproductive ecology: patterns and strategies.

[14] Raikov IB (1995). Meiosis in protists: recent advances and persisting problems. Eur J Protistol..

[15] Corley LS, Blankenship JR, Moore AJ (2001). Genetic variation and asexual reproduction in the facultatively parthenogenetic cockroach *Nauphoeta cinerea*: implications for the evolution of sex. J Evol Biol..

[16] Eads BD, Colbourne JK, Bohuski E, Andrews J (2007). Profiling sex-biased gene expression during parthenogenetic reproduction in Daphnia pulex. BMC Genomics..

[17] Keeling PJ, Palmer JD (2008). Horizontal gene transfer in eukaryotic evolution. Nat Rev Genet.

[18] Funk DH, Sweeney BW, Jackson JK (2010). Why stream mayflies can reproduce without males but remain bisexual: a case of lost genetic variation. J North Am Benthol Soc..

[19] da Barbiano LA, Gompert Z, Aspbury AS, Gabor CR, Nice CC (2013). Population genomics reveals a possible history of backcrossing and recombination in the gynogenetic fish Poecilia formosa. PNAS..

[20] Warren WC, García-Pérez R, Xu S, Lampert KP, Chalopin D, Stöck M, Loewe L, Lu Y, Kuderna L, Minx P, Montague MJ (2018). Clonal polymorphism and high heterozygosity in the celibate genome of the Amazon molly. Nat Ecol Evol..

[21] Bogart JP, Bi K, Fu J, Noble DW, Niedzwiecki J (2007). Unisexual salamanders (genus *Ambystoma*) present a new reproductive mode for eukaryotes. Genome..

[22] Bi K, Bogart JP (2010). Probing the meiotic mechanism of intergenomic exchanges by genomic in situ hybridization on lampbrush chromosomes of unisexual Ambystoma (Amphibia: Caudata). Chromosome Res..

[23] Avise JC (2015). Evolutionary perspectives on clonal reproduction in vertebrate animals. PNAS..

[24] Tarkhnishvili D, Murtskhvaladze M, Anderson CL (2017). Coincidence of genotypes at two loci in two parthenogenetic rock lizards: how backcrosses might trigger adaptive speciation. Biol J Linn Soc..

[25] Kearney M, Fujita MK, Ridenour J, Schön I, Martens K, Dijk P (2009). Lost sex in the reptiles: constraints and correlations. Lost sex.

[26] Bezy RL, Camarillo JL (2002). Systematics of xantusiid lizards of the genus. Lepidophyma. Contrib Sci..

[27] Vrijenhoek RC, Parker ED, Schön I, Martens K, Dijk P (2009). Geographical parthenogenesis: general purpose genotypes and frozen niche variation. Lost sex.

[28] Schön I, Martens K, van Dijk P (2009). Lost sex. The evolutionary biology of parthenogenesis.

[29] Pennock LA (1965). Triploidy in parthenogenetic species of the teiid lizard, genus Cnemidophorus. Science..

[30] Wright JW, Spolsky C, Brown WM (1983). The origin of the parthenogenetic lizard Cnemidophorus laredoensis inferred from mitochondrial DNA analysis. Herpetologica..

[31] Wright JW, Vitt LJ (1994). Biology of whiptail lizards (genus Cnemidophorus). Syst Biol..

[32] Darevsky IS, Kupriyanova LA, Uzzell T, Gans C, Billet F (1985). Parthenogenesis in reptiles. Biology of the reptilia.

[33] Moritz C, Brown WM, Densmore LD, Wright JW, Vyas D, Donnellan S, Adams M, Baverstock P, Dawley RM, Bogart JP (1989). Genetic diversity and the dynamics of hybrid parthenogenesis in Cnemidophorus (Teiidae) and Heteronotia (Gekkonidae). Evolution and ecology of unisexual vertebrates.

[34] Moritz C, Uzzell T, Spolsky C, Hotz H, Darevsky I, Kupriyanova L, Danielyan F (1992). The material ancestry and approximate age of parthenogenetic species of Caucasian rock lizards (*Lacerta*: Lacertidae). Genetica.

[35] Tarkhnishvili D. Evolutionary history, habitats, diversification, and speciation in Caucasian rock lizards. In Jenkins OP, ed. Advances in Zoology Research. 2012;2: p. 79-120.

[36] Freitas S, Rocha S, Campos J, Ahmadzadeh F, Corti C, Sillero N, Ilgaz Ç, Kumlutaş Y, Arakelyan M, Harris DJ, Carretero MA (2016). Parthenogenesis through the ice ages: a biogeographic analysis of Caucasian rock lizards (genus Darevskia). Mol Phylogenet Evol..

[37] Freitas SN, Harris DJ, Sillero N, Arakelyan M, Butlin RK, Carretero MA (2019). The role of hybridisation in the origin and evolutionary persistence of vertebrate parthenogens: a case study of Darevskia lizards. Heredity..

[38] Lantz LA, Cyrén O (1936). Contribution à la connaissance de Lacerta saxicola Eversmann. Bulletin de la Société zoologique de France..

[39] Darevsky IS (1957). Systematics and ecology of rock lizards (Lacerta saxicola Eversmann) in Armenia. Zool Sb AN Armenia SSR..

[40] Darevsky IS. Skal’nye yashcheritsy Kavkaza. Leningrad: Nauka (Translated as: Rock lizards of the Caucasus. New Delhi: Indian National Scientific Documentation Centre). 1967.

[41] Darevsky IS, Danielyan F (1977). Lacerta uzzelli sp. nov.(Sauria, Lacertidae)–a new parthenogenetic species of rock lizard from eastern Turkey. Trudy Zoological Institute. Leningrad..

[42] Schmidtler JF, Eiselt JO, Darevsky IS (1994). Untersuchungen an Felseidechsen (Lacerta-saxicola-Gruppe) in der östlichen Türkei: 3. Zwei neue parthenogenetische Arten. Salamandra..

[43] Roquet C, Lavergne S, Thuiller W. One tree to link them all: A phylogenetic dataset for the European tetrapoda. PLoS Curr. 2014;6. 10.1371/currents.tol.5102670fff8aa5c918e78f5592790e48. 10.1371/currents.tol.5102670fff8aa5c918e78f5592790e48 PMC4322008 25685620

[44] Zheng Y, Wiens JJ (2016). Combining phylogenomic and supermatrix approaches, and a time-calibrated phylogeny for squamate reptiles (lizards and snakes) based on 52 genes and 4162 species. Mol Phylogenet Evol..

[45] Murphy RW, Fu J, Macculloch RD, Darevsky IS, Kupriyanova LA (2000). A fine line between sex and unisexuality: the phylogenetic constraints on parthenogenesis in lacertid lizards. Zool J Linn Soc..

[46] Tarkhnishvili D, Murtskhvaladze M, Gavashelishvili A (2013). Speciation in Caucasian lizards: climatic dissimilarity of the habitats is more important than isolation time. Biol J Linn Soc..

[47] Parker ED, Walker JM, Paulissen MA, Dawley RM, Bogart JP (1989). Clonal diversity in Cnemidophorus: ecological and morphological consequences. Evolution and ecology of unisexual vertebrates.

[48] Fu J, MacCulloch RD, Murphy RW, Darevsky IS, Tuniyev BS (2000). Allozyme variation patterns and multiple hybridization origins: clonal variation among four sibling parthenogenetic Caucasian rock lizards. Genetica..

[49] MacCulloch RD, Murphy RW, Kupriyanova LA, Darevsky IS, Danielyan FD (1995). Clonal variation in the parthenogenetic rock lizard Lacerta armeniaca. Genome..

[50] Fu J, Murphy RW, Darevsky IS (1999). Limited genetic variation in Lacerta mixta and its parthenogenetic daughter species: evidence from cytochrome b and ATPase 6 gene DNA sequences. Genetica..

[51] Murphy RW, Darevsky IS, MacCulloch RD, Fu J, Kupriyanova LA, Upton DE, Danielyan F (1997). Old age, multiple formations or genetic plasticity? Clonal diversity in the uniparental Caucasian rock lizard, Lacerta dahli. Genetica..

[52] Kan NG, Martirosyan IA, Ryskov AP, Tokarskaya ON, Petrosyan VG, Grechko VV, Darevsky IS, Danielyan FD, Ryabinin DM (1998). Genomic polymorphism of mini-and microsatellite loci of the parthenogenic Lacerta dahli revealed by DNA fingerprinting. Mol Biol..

[53] Ryskov AP, Osipov FA, Omelchenko AV, Semyenova SK, Girnyk AE, Korchagin VI, Vergun AA, Murphy RW. The origin of multiple clones in the parthenogenetic lizard species Darevskia rostombekowi. PloSone. 2017;12. 10.1371/journal.pone.0185161. 10.1371/journal.pone.0185161 PMC5607197 28931071

[54] Vergun AA, Martirosyan IA, Semyenova SK, Omelchenko AV, Petrosyan VG, Lazebny OE, Tokarskaya ON, Korchagin VI, Ryskov AP. Clonal diversity and clone formation in the parthenogenetic Caucasian rock lizard Darevskia dahli. PloSone. 2014;9. 10.1371/journal.pone.0091674. 10.1371/journal.pone.0091674 PMC3950254 24618670

[55] Girnyk AE, Vergun AA, Semyenova SK, Guliaev AS, Arakelyan MS, Danielyan FD, Martirosyan IA, Murphy RW, Ryskov AP (2018). Multiple interspecific hybridization and microsatellite mutations provide clonal diversity in the parthenogenetic rock lizard Darevskia armeniaca. BMC genomics..

[56] Tokarskaya ON, Martirosyan IA, Badaeva TN, Malysheva DN, Korchagin VI, Darevsky IS, Danielyan FD, Ryskov AP (2004). Instability of (GATA) n microsatellite loci in the parthenogenetic Caucasian rock lizard Darevskia unisexualis (Lacertidae). Mol Genet Genom..

[57] Badaeva TN, Malysheva DN, Korchagin VI, Ryskov AP. Genetic variation and de novo mutations in the parthenogenetic Caucasian rock lizard Darevskia unisexualis. PLoS One. 2008;3(7). 10.1371/journal.pone.0002730. 10.1371/journal.pone.0002730 PMC2447159 18648496

[58] Darevsky IS, Kulikova VN (1964). Natural triploidy in polymorphic group of Caucasian rock lizards (Lacerta saxicola Eversmann) as result of hybridization of bisexual with parthenogenetic forms of these species. Doklady Akademii Nauk SSSR..

[59] Darewskii IS, Kulikova VN (1961). Naturliche Parthenogenese in der polymorphen Gruppe der kaukasischen Felseidechse (Lacerta saxicola Eversmann). Zool Jahrb: Abt Systematik Okologie Geogr Tiere..

[60] Darevsky IS, Uzell TM, Kupriyanova LA, Danielyan FD (1973). Triploid hybrid males in sympatric populations of some parthenogenetic and bisexual species of rock lizards of the genus Lacerta. Bull Mosc Soc Nat..

[61] Darevsky IS, Danielyan FD (1968). Diploid and triploid progeny arising from natural mating of parthenogenetic Lacerta armeniaca and L. unisexualis with bisexual L. saxicola valentini. J Herpetol..

[62] Arakelyan M, Danielyan F, Stepanyan I (2008). Hybrids of Darevskia valentini, D. armeniaca and D. unisexualis from a sympatric population in Armenia. Amphibia-Reptilia..

[63] Spangenberg V, Arakelyan M, Galoyan E, Matveevsky S, Petrosyan R, Bogdanov Y, Danielyan F, Kolomiets O (2017). Reticulate evolution of the rock lizards: meiotic chromosome dynamics and spermatogenesis in diploid and triploid males of the genus Darevskia. Genes..

[64] Danielyan F, Arakelyan M, Stepanyan I. The progress of microevolution in hybrids of rock lizards of genus Darevskia. Biol J Armenia. 2008;60:147–56.

[65] Kupriyanova LA, Dawley RM, Bogart JP (1989). Cytogenetic evidence for genome interaction in hybrid lacertid lizards. Evolution and ecology of unisexual vertebrates.

[66] Murtskhvaladze M, Tarkhnishvili D, Anderson CL, Kotorashvili A. Phylogeny of Caucasian rock lizards (Darevskia) and other true lizards based on mitogenome analysis: Optimisation of the algorithms and gene selection. PlosOne. 2020;15(6). 10.1371/journal.pone.0233680. 10.1371/journal.pone.0233680 PMC7279592 32511235

[67] Gabelaia M, Tarkhnishvili D, Murtskhvaladze M (2015). Phylogeography and morphological variation in a narrowly distributed Caucasian rock lizard, Darevskia mixta. Amphibia-Reptilia..

[68] Petit RJ, El Mousadik A, Pons O (1998). Identifying populations for conservation on the basis of genetic markers. Conserv Biol..

[69] Wahlund S (1928). Zusammensetzung von Population und Korrelationserscheinung vom Standpunkt der Vererbungslehre aus betrachtet. Hereditas.

[70] Lutes AA, Neaves WB, Baumann DP, Wiegraebe W, Baumann P (2010). Sister chromosome pairing maintains heterozygosity in parthenogenetic lizards. Nature.

[71] Bogart JP, Bi K (2013). Genetic and genomic interactions of animals with different ploidy levels. Cytogenet Genome Res..

[72] Uzzell T, Darevsky IS (1975). Biochemical evidence for the hybrid origin of the parthenogenetic species of the Lacerta saxicola complex (Sauria: Lacertidae), with a discussion of some ecological and evolutionary implications. Copeia.

[73] Darevsky IS (1995). Epistandard evolution and hybridogenous speciation in reptiles. Zhurnal Obshchei Biologii.

[74] Tarkhnishvili D, Gavashelishvili A, Avaliani A, Murtskhvaladze M, Mumladze L (2010). Unisexual rock lizard might be outcompeting its bisexual progenitors in the Caucasus. Biol J Linn Soc..

[75] Gaggiotti OE (1994). An ecological model for the maintenance of sex and geographic parthenogenesis. J Theor Biol..

[76] Estoup A, Jarne P, Cornuet JM (2002). Homoplasy and mutation model at microsatellite loci and their consequences for population genetics analysis. Mol Ecol..

[77] Amos W (2016). Heterozygosity increases microsatellite mutation rate. Biol Lett..

[78] Gibbs HL, Denton RD (2016). Cryptic sex? Estimates of genome exchange in unisexual mole salamanders (A mbystoma sp.). Mol Ecol..

[79] Spangenberg V, Arakelyan M, de Bello Cioffi M, Liehr T, Al-Rikabi A, Martynova E, Danielyan F, Stepanyan I, Galoyan E, Kolomiets O. cytogenetic mechanisms of unisexuality in rock lizards. Sci Rep. 2020;10:1-4. doi: 10.5281/zenodo.3611475. 10.1038/s41598-020-65686-7 PMC7250862 32457493

[80] Lamatsch DK, Stöck M, Schön I, Martens K, Dijk P (2009). Sperm-dependent parthenogenesis and hybridogenesis in teleost fishes. Lost sex.

[81] Betto-Colliard C, Sermier R, Litvinchuk S, Perrin N, Stöck M (2015). Origin and genome evolution of polyploid green toads in Central Asia: evidence from microsatellite markers. Heredity.

[82] Betto-Colliard C, Hofmann S, Sermier R, Perrin N, Stöck M (2018). Profound genetic divergence and asymmetric parental genome contributions as hallmarks of hybrid speciation in polyploid toads. Proc Roy Soc B-Biol Sci..

[83] Parker ED, Selander RK (1976). The organization of genetic diversity in the parthenogenetic lizard Cnemidophorus tesselatus. Genetics..

[84] Taylor HL, Cole CJ, Hardy LM, Dessauer HC, Townsend CR, Walker JM, Cordes JE (2001). Natural hybridization between the teiid lizards Cnemidophorus tesselatus (parthenogenetic) and C. tigris marmoratus (bisexual): assessment of evolutionary alternatives. Am Mus Novit..

[85] Lowcock LA, Bogart JP (1989). Electrophoretic evidence for multiple origins of triploid forms in the Ambystoma laterale–jeffersonianum complex. Canadian J Zool..

[86] Bogart JP (2019). A family study to examine clonal diversity in unisexual salamanders (genus Ambystoma). Genome..

[87] Morishima K, Yoshikawa H, Arai K (2008). Meiotic hybridogenesis in triploid Misgurnus loach derived from a clonal lineage. Heredity..

[88] Griffiths AJ, Gelbart WM, Lewontin RC, Miller JH. Modern Genetic Analysis: Integrating Genes and Genomes. W.H. Freeman and Company; 2002.

[89] Spangenberg V, Arakelyan M, Galoyan E, Pankin M, Petrosyan R, Stepanyan I, Grishaeva T, Danielyan F, Kolomiets O (2019). Extraordinary centromeres: differences in the meiotic chromosomes of two rock lizards species Darevskia portschinskii and Darevskia raddei. PeerJ..

[90] Flot JF, Hespeels B, Li X, Noel B, Arkhipova I, Danchin EG, Hejnol A, Henrissat B, Koszul R, Aury JM, Barbe V (2013). Genomic evidence for ameiotic evolution in the bdelloid rotifer Adineta vaga. Nature.

[91] Lokki J (1976). Genetic polymorphism and evolution in parthenogenetic animals: VII. The amount of heterozygosity in diploid populations. Hereditas..

[92] Orr HA, Turelli M (2001). The evolution of postzygotic isolation: accumulating Dobzhansky‐Muller incompatibilities. Evolution..

[93] Wu CI, Palopoli MF (1994). Genetics of postmating reproductive isolation in animals. Ann Rev Genet..

[94] Degenhardt WG, Painter CW, Price AH (1996). Amphibians and Reptiles of New Mexico. Univ New Mexico Press Albuquerque..

[95] Reeder TW, Cole CJ, Dessauer HC (2002). Phylogenetic relationships of whiptail lizards of the genus Cnemidophorus (Squamata: Teiidae): a test of monophyly, reevaluation of karyotypic evolution, and review of hybrid origins. Am Mus Novit..

[96] Lutes AA, Baumann DP, Neaves WB, Baumann P (2011). Laboratory synthesis of an independently reproducing vertebrate species. PNAS..

[97] Cole CJ, Hardy LM, Dessauer HC, Taylor HL, Townsend CR (2010). Laboratory hybridization among North American whiptail lizards, including Aspidoscelis inornata arizonae× A. tigris marmorata (Squamata: Teiidae), ancestors of unisexual clones in nature. Am Mus Novit..

[98] DNeasy Blood & Tissue Handbook. http://www.bea.ki.se/documents/EN-DNeasy%20handbook.pdf. Accessed 14 Sept 2020.

[99] Kumar S, Stecher G, Li M, Knyaz C, Tamura K (2018). MEGA X: molecular evolutionary genetics analysis across computing platforms. Mol Biol Evol..

[100] Bandelt HJ, Forster P, Röhl A (1999). Median-joining networks for inferring intraspecific phylogenies. Mol Biol Evol..

[101] Omelchenko AV, Korchagin VI, Sevast’yanova GA, Ryskov AP, Tokarskaya ON (2009). Molecular genetic characteristic of dinucleotide microsatellite loci in parthenogenetic lizards Darevskia unisexualis. Russ J Genet..

[102] Korchagin VI, Badaeva TN, Tokarskaya ON, Martirosyan IA, Darevsky IS, Ryskov AP (2007). Molecular characterization of allelic variants of (GATA) n microsatellite loci in parthenogenetic lizards Darevskia unisexualis (Lacertidae). Gene..

[103] Waits LP, Luikart G, Taberlet P (2001). Estimating the probability of identity among genotypes in natural populations: cautions and guidelines. Mol Ecol..

[104] Miller CR, Joyce P, Waits LP (2002). Assessing allelic dropout and genotype reliability using maximum likelihood. Genetics..

[105] Pompanon F, Bonin A, Bellemain E, Taberlet P (2005). Genotyping errors: causes, consequences and solutions. Nat Rev Genet.

[106] Jombart T (2008). adegenet: a R package for the multivariate analysis of genetic markers. Bioinformatics..

[107] Goudet J (2005). Hierfstat, a package for R to compute and test hierarchical F‐statistics. Mol Ecol Notes..

[108] Adamack AT, Gruber B (2014). PopGenReport: simplifying basic population genetic analyses in R. Methods Ecol Evol..

[109] Prim RC (1957). Shortest connection networks and some generalizations. Bell Syst Tech J..

[110] Kamvar ZN, Tabima JF, Grünwald NJ (2014). Poppr: an R package for genetic analysis of populations with clonal, partially clonal, and/or sexual reproduction. PeerJ..

[111] Kamvar ZN, Brooks JC, Grünwald NJ (2015). Novel R tools for analysis of genome-wide population genetic data with emphasis on clonality. Front Genet.

[112] Csardi G, Nepusz T (2006). The igraph software package for complex network research. InterJ Complex Syst..

[113] Bruvo R, Michiels NK, D’souza TG, Schulenburg H (2004). A simple method for the calculation of microsatellite genotype distances irrespective of ploidy level. Mol Ecol..

[114] Falush D, Stephens M, Pritchard JK (2003). Inference of population structure using multilocus genotype data: linked loci and correlated allele frequencies. Genetics..

[115] Evanno G, Regnaut S, Goudet J (2005). Detecting the number of clusters of individuals using the software STRUCTURE: a simulation study. Mol Ecol..

[116] Pritchard JK, Wen X, Falush D (2010). Documentation for STRUCTURE software, version 2.3.

[117] Earl DA (2012). STRUCTURE HARVESTER: a website and program for visualizing STRUCTURE output and implementing the Evanno method. Conserv Genet Resour..

[118] Jackson DA (1993). Stopping rules in principal components analysis: a comparison of heuristical and statistical approaches. Ecology..

[119] Murgia C, Pritchard JK, Kim SY, Fassati A, Weiss RA (2006). Clonal origin and evolution of a transmissible cancer. Cell..

